# Metabolic Mechanism and Physiological Role of Glycerol 3-Phosphate in Pseudomonas aeruginosa PAO1

**DOI:** 10.1128/mbio.02624-22

**Published:** 2022-10-11

**Authors:** Yidong Liu, Wenxuan Sun, Liting Ma, Rong Xu, Chunyu Yang, Ping Xu, Cuiqing Ma, Chao Gao

**Affiliations:** a State Key Laboratory of Microbial Technology, Shandong University, Qingdao, People’s Republic of China; b State Key Laboratory of Microbial Metabolism, Shanghai Jiao Tong University, Shanghai, People’s Republic of China; c School of Life Sciences and Biotechnology, Shanghai Jiao Tong University, Shanghai, People’s Republic of China; Korea Advanced Institute of Science and Technology

**Keywords:** *Pseudomonas aeruginosa*, glycerol, glycerol 3-phosphate, glycerol 3-phosphate dehydrogenase, glycerol 3-phosphate phosphatase

## Abstract

Pseudomonas aeruginosa is an important opportunistic pathogen that is lethal to cystic fibrosis (CF) patients. Glycerol generated during the degradation of phosphatidylcholine, the major lung surfactant in CF patients, could be utilized by P. aeruginosa. Previous studies have indicated that metabolism of glycerol by this bacterium contributes to its adaptation to and persistence in the CF lung environment. Here, we investigated the metabolic mechanisms of glycerol and its important metabolic intermediate glycerol 3-phosphate (G3P) in P. aeruginosa PAO1. We found that G3P homeostasis plays an important role in the growth and virulence factor production of P. aeruginosa PAO1. The G3P accumulation caused by the mutation of G3P dehydrogenase (GlpD) and exogenous glycerol led to impaired growth and reductions in pyocyanin synthesis, motilities, tolerance to oxidative stress, and resistance to kanamycin. Transcriptomic analysis indicates that the growth retardation caused by G3P stress is associated with reduced glycolysis and adenosine triphosphate (ATP) generation. Furthermore, two haloacid dehalogenase-like phosphatases (PA0562 and PA3172) that play roles in the dephosphorylation of G3P in strain PAO1 were identified, and their enzymatic properties were characterized. Our findings reveal the importance of G3P homeostasis and indicate that GlpD, the key enzyme for G3P catabolism, is a potential therapeutic target for the prevention and treatment of infections by this pathogen.

## INTRODUCTION

Pseudomonas aeruginosa is an opportunistic Gram-negative pathogen that infects a broad range of hosts, including plants, animals, and humans (1). It is recognized as the primary cause of morbidity and mortality in patients with cystic fibrosis (CF) (2). The considerable metabolic versatility of P. aeruginosa enables it to adapt to and persist in the CF lung environment (3). Phosphatidylcholine, the major lung surfactant in CF patients, can be cleaved into phosphorylcholine, fatty acids, and glycerol by P. aeruginosa-produced phospholipase C and lipases (4). Importantly, it has been demonstrated that glycerol metabolic genes are upregulated in P. aeruginosa isolates obtained from patients with CF (4), which indicates that the metabolism of glycerol may contribute to the persistence of P. aeruginosa in the CF lung environment.

During aerobic microbial metabolism, glycerol is initially phosphorylated to glycerol 3-phosphate (G3P) by the adenosine triphosphate (ATP)-dependent glycerol kinase (GlpK). Subsequently, G3P dehydrogenase (GlpD) catalyzes the dehydrogenation of G3P to yield dihydroxyacetone phosphate (DHAP), which undergoes isomerization to glyceraldehyde-3-phosphate prior to entering the gluconeogenic or glycolytic pathway (5, 6). In addition, G3P can be acylated by acyl-CoA to initiate the biosynthesis of membrane lipids (7) and storage lipids (8) in microbes. However, the generation of excessive G3P may also be toxic, and indeed, as early as 1965, the accumulation of G3P was reported to cause the growth stasis of Escherichia coli (9). In some glycerol-producing microbes, such as Saccharomyces cerevisiae (10) and Corynebacterium glutamicum (11), glycerol 3-phosphate phosphatase (G3PP), which dephosphorylates G3P to yield glycerol, has been identified.

Given its impressive intrinsic resistance, potential to acquire resistance to current antibiotics, and deployment of an arsenal of virulence factors, the prevention of P. aeruginosa infections presents a considerable challenge in health care settings (2, 12). Accordingly, there exists an urgent need to develop new therapeutic strategies by which to combat multidrug-resistant P. aeruginosa. Many recent studies have indicated that there is a close association between physiological metabolism and bacterial sensitivity to antibiotics and their virulence (13–15). Thus, it is plausible that inhibiting specific enzymes in bacterial metabolic pathways may also contribute to the control of drug-resistant bacteria. For example, Puckett et al. (16) have demonstrated that deletion of malate synthase induces the accumulation of the toxic metabolite glyoxylate during the utilization of host fatty acids by Mycobacterium tuberculosis, thereby reducing its growth and persistence in acute and chronic mouse infections. Accordingly, malate synthase was proposed as a potential drug target in cases of M. tuberculosis infections. Similarly, promysalin, which has recently been identified as an inhibitor of the succinate dehydrogenase of P. aeruginosa, is considered to be a promising “narrow-spectrum” antibiotic for the treatment of P. aeruginosa infections (17). Given the putative role of glycerol metabolism in the adaptation of P. aeruginosa to the CF lung environment, we speculate that the proteins associated with the metabolism of glycerol or with its phosphorylated product G3P may serve as antibiotic targets for the treatment of P. aeruginosa infections.

In the present study, we seek to elucidate the mechanisms underlying the synthesis, degradation, and transport of G3P in P. aeruginosa type strain PAO1. We demonstrate the importance of G3P homeostasis for its growth and virulence factor production. The decreased transcription of genes in the Entner-Doudoroff (ED) pathway and impaired ATP production may be associated with the growth defects induced by G3P accumulation. In addition, we identifiy PA0562 and PA3172 as the G3PPs that mediate the dephosphorylation of this toxic metabolite. These findings indicate that the G3P catabolic enzyme GlpD could serve as a potential therapeutic target by which to control P. aeruginosa infections.

## RESULTS

### Identification of G3P metabolism-related genes in P. aeruginosa PAO1.

**FIG 1:**
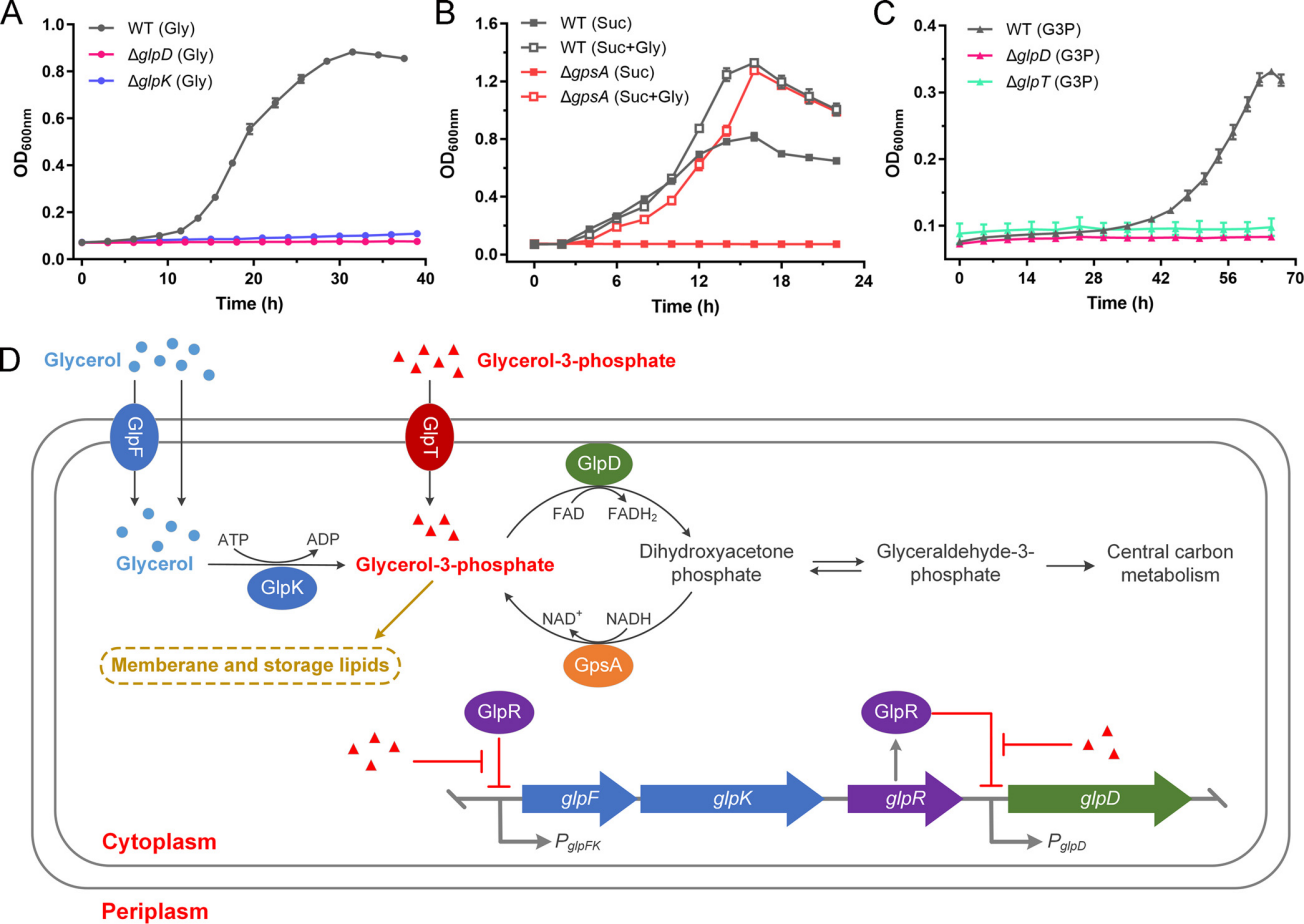
Synthesis, degradation, and transport mechanisms of glycerol 3-phosphate (G3P) in P. aeruginosa PAO1. (A) Growth of P. aeruginosa PAO1 (black line), Δ*glpK* (blue line), and Δ*glpD* (pink line) with 20 mM glycerol as the sole carbon source. (B) Growth of P. aeruginosa PAO1 (black line) and Δ*gpsA* (red line) with 20 mM succinate (Suc) or 20 mM succinate and 20 mM glycerol (Suc + Gly) as the carbon sources. (C) Growth of P. aeruginosa PAO1 (black line), Δ*glpD* (pink line), and Δ*glpT* (green line) with 10 mM G3P as the sole carbon source. (D) Schematic representation of G3P metabolism in P. aeruginosa PAO1. G3P can be imported from the extracellular environment, derived from glycerol phosphorylation, or synthesized via the reduction of dihydroxyacetone phosphate. Then, G3P will be fluxed into membrane and storage lipid biosynthesis or into the central carbon metabolism. GlpR represses the expression of glycerol metabolic regulon and uses G3P as its effector. All data shown are the average values of three independent experiments ± the standard deviation (SD).

In P. aeruginosa PAO1, the genes involved in glycerol metabolism, including *glpF* (glycerol facilitator, *PA3581*), *glpK* (glycerol kinase, *PA3582*), *glpR* (glycerol regulon repressor, *PA3583*), and *glpD* (G3P dehydrogenase, *PA3584*), are arranged in a genomic cluster. These genes have provisionally been identified or annotated in previous studies (1, 18–20). In the present study, we initially examined their roles in the metabolism of glycerol by P. aeruginosa PAO1. Neither the Δ*glpD* nor the Δ*glpK* mutants grew in minimal salt medium (MSM) containing 20 mM glycerol as the sole carbon source (Fig. 1A). The loss of *glpF* was found to affect the growth and glycerol utilization of P. aeruginosa PAO1 (Δ*glpF*) only at a low glycerol concentration (2 mM) (Fig. S1A and B). Conversely, the knockout of *glpR* enabled P. aeruginosa PAO1 (Δ*glpR*) to grow more rapidly than the wild-type strain when glycerol was the sole carbon source (Fig. S1C).

10.1128/mbio.02624-22.1 FIG S1 Functional identification of glycerol or G3P metabolic genes in P. aeruginosa PAO1. (A and B) Growth of P. aeruginosa PAO1 (black line) and Δ*glpF* (green line) with either 20 mM glycerol (A) or 2 mM glycerol (B) as the sole carbon source. (C) Growth of P. aeruginosa PAO1 (black line) and Δ*glpR* (orange line) with 20 mM glycerol as the sole carbon source. (D and E) G3P prevents GlpR binding to the *glpFK* (D) and *glpD* (E) promoter regions. DNA probes (3.5 nM) were incubated with or without 500 nM GlpR. G3P or glycerol at the concentration of 10 mM was added as indicated above the corresponding lanes. Lane M, molecular weight markers. A 217-bp internal fragment of *glpD* (3.5 nM) was used as a negative-control probe. (F) Growth of P. aeruginosa PAO1 (black line) and Δ*gpsA* (red line) with 20 mM glucose (Glu) or 20 mM glucose and glycerol (Glu + Gly) as the carbon sources. All data shown are the average values of three independent experiments ± SD. Download FIG S1, TIF file, 1.2 MB. Copyright © 2022 Liu et al. 2022 Liu et al. https://creativecommons.org/licenses/by/4.0/ This content is distributed under the terms of the Creative Commons Attribution 4.0 International license.

We established that GlpR represses the utilization of glycerol by P. aeruginosa PAO1 by binding to the promoters of *glpFK* and *glpD* (Fig. S1D and E), as has been observed in P. putida KT2440 (21). Interestingly, it uses G3P rather than glycerol as the effector to induce the expression of *glpFK* and *glpD* (Fig. S1D and E). We also examined the metabolism of G3P by P. aeruginosa PAO1. In addition to the phosphorylation of glycerol, G3P can be generated via DHAP reduction. The biosynthetic G3P dehydrogenase (GpsA), which catalyzes this reduction for *de novo* G3P biosynthesis *in vivo*, has previously been identified in E. coli (22, 23). The orthologous protein of GpsA has also been annotated in the P. aeruginosa PAO1 genome. It was found that the knockout of *gpsA* (*PA1614*) results in a G3P auxotrophic phenotype, which could be complemented by the provision of exogenous glycerol (Fig. 1B; Fig. S1F). It was also found that P. aeruginosa PAO1 could grow in MSM containing 10 mM G3P as the sole carbon source (Fig. 1C). The G3P transporter (GlpT) has previously been identified in P. aeruginosa (24). Furthermore, the knockout of *glpT* (*PA5235*) or *glpD* impaired the ability of P. aeruginosa PAO1 to utilize G3P (Fig. 1C), thereby demonstrating the role of GlpT in G3P transport and the role of GlpD in G3P catabolism.

From these results, we can summarize the synthesis, degradation, and transport mechanisms of G3P in P. aeruginosa PAO1. As shown in Fig. 1D, the production of G3P can be achieved via the phosphorylation of glycerol by GlpK and the reduction of DHAP by GpsA, respectively. In addition, extracellular G3P can enter cells via GlpT-mediated transport, whereas the catabolism of G3P depends on GlpD-catalyzed dehydrogenation to produce DHAP.

### Mutation of *glpD* affects production of pyocyanin and exopolysaccharides, twitching motility, and oxidative stress tolerance.

**FIG 2:**
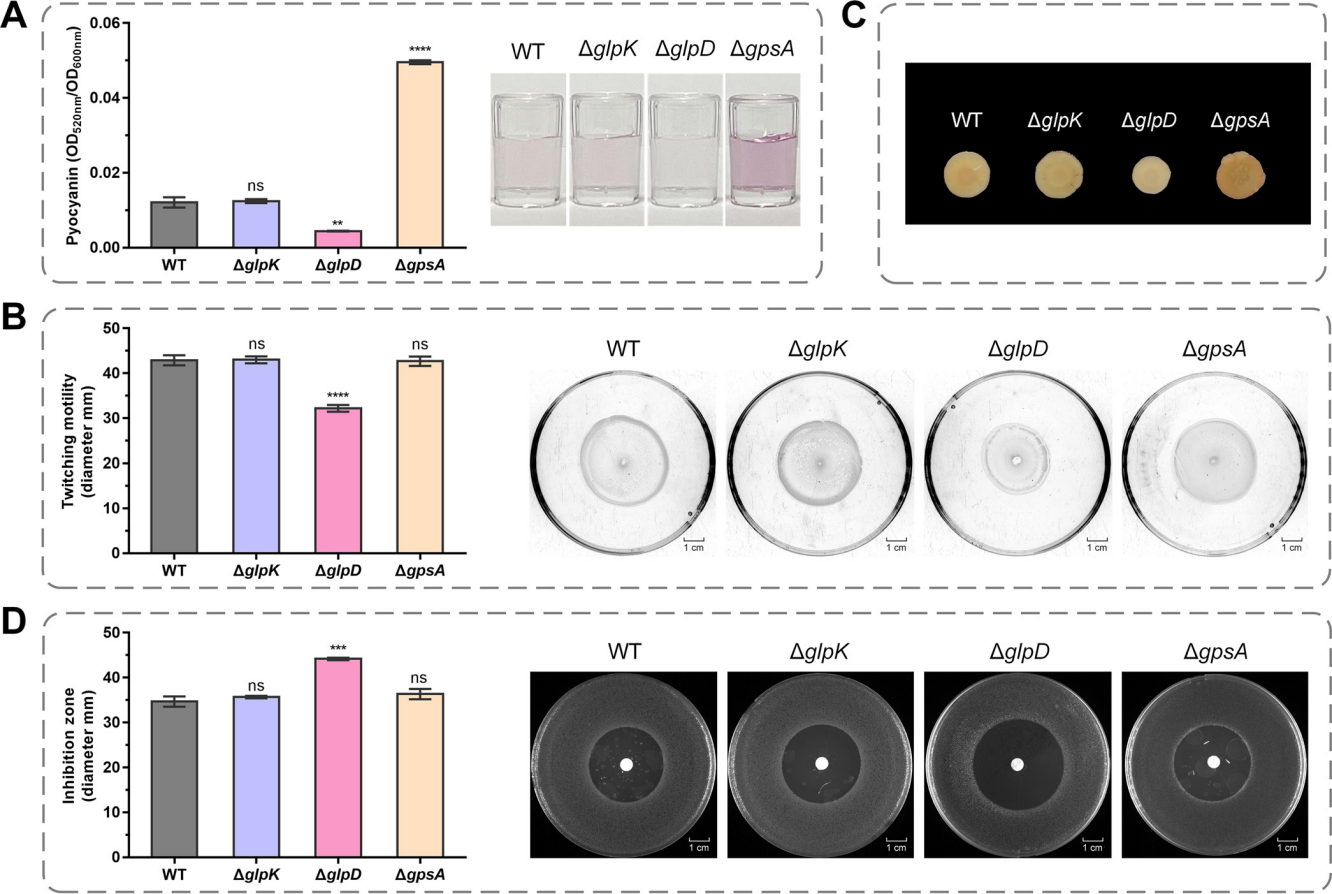
Pyocyanin, exopolysaccharides, twitching motility, and oxidative stress tolerance of P. aeruginosa PAO1 and its derivatives. (A) Spectrophotometric quantitation of pyocyanin produced by P. aeruginosa PAO1 and its derivatives at stationary-phase. Pyocyanin production was also indicated by photographs of the acidified extracts of the cultures of P. aeruginosa PAO1 and its derivatives with chloroform. The pink color is proportional to the amount of pyocyanin. (B) Diameters of subsurface twitching motility zones. Twitching motility was also indicated by photographs of P. aeruginosa PAO1 and its derivatives on 1% agar plates. (C) Colony morphology on Congo red plates. (D) Diameters and photographs of bacteriostatic zones generated by 30% (wt/wt) H_2_O_2_. All strains were grown with Luria-Bertani (LB) medium. All data shown are the average values of three independent experiments ± SD. Two-tailed *P* values were determined using unpaired *t* tests. **, *P* < 0.01; ***, *P* < 0.001; ****, *P* < 0.0001; ns, no significant difference (*P* ≥ 0.05).

GlpR uses G3P instead of glycerol as its effector to regulate glycerol and G3P catabolism (Fig. S1D and E), thereby implying that intracellular G3P, rather than glycerol, is involved in maintaining the cellular homeostasis of P. aeruginosa PAO1. To characterize the G3P metabolism of P. aeruginosa PAO1, we cultured the wild-type strain and mutants related to G3P metabolism in Luria-Bertani (LB) medium. Then, the pyocyanin production, twitching motility, exopolysaccharide generation, and oxidative stress tolerance of P. aeruginosa PAO1, Δ*glpD*, Δ*glpK*, and Δ*gpsA* were assayed. Pyocyanin is a vital virulence factor produced by P. aeruginosa (3). As shown in Fig. 2A, there was a clear reduction in the pyocyanin production of the Δ*glpD* mutant, whereas we detected a significant increase in that of Δ*gpsA*. The type IV pili-mediated twitching motility of P. aeruginosa is necessary for bacterial adhesion and for the initiation of infection in CF patients (25). As shown in Fig. 2B, the knockout of either *glpD* or *gpsA* resulted in a reduction in twitching motility. The cells of P. aeruginosa often reside in exopolysaccharide-enclosed biofilms, which are assumed to confer enhanced resistance to antibiotics (26). To quantify the exopolysaccharides produced by P. aeruginosa PAO1 and its G3P metabolism-related mutants, we performed a Congo red binding assay (27). The results showed that fewer exopolysaccharides were produced by the Δ*glpD* mutant (Fig. 2C). Hydrogen peroxide (H_2_O_2_) is an oxidizing agent produced by the host immune system (28). H_2_O_2_ sensitivity assays revealed that the knockout of *glpD* reduced the tolerance of P. aeruginosa PAO1 to oxidative stress (Fig. 2D).

### G3P accumulation leads to reductions in growth, pyocyanin production, motilities, and oxidative stress tolerance, as well as antibiotic susceptibility.

**FIG 3:**
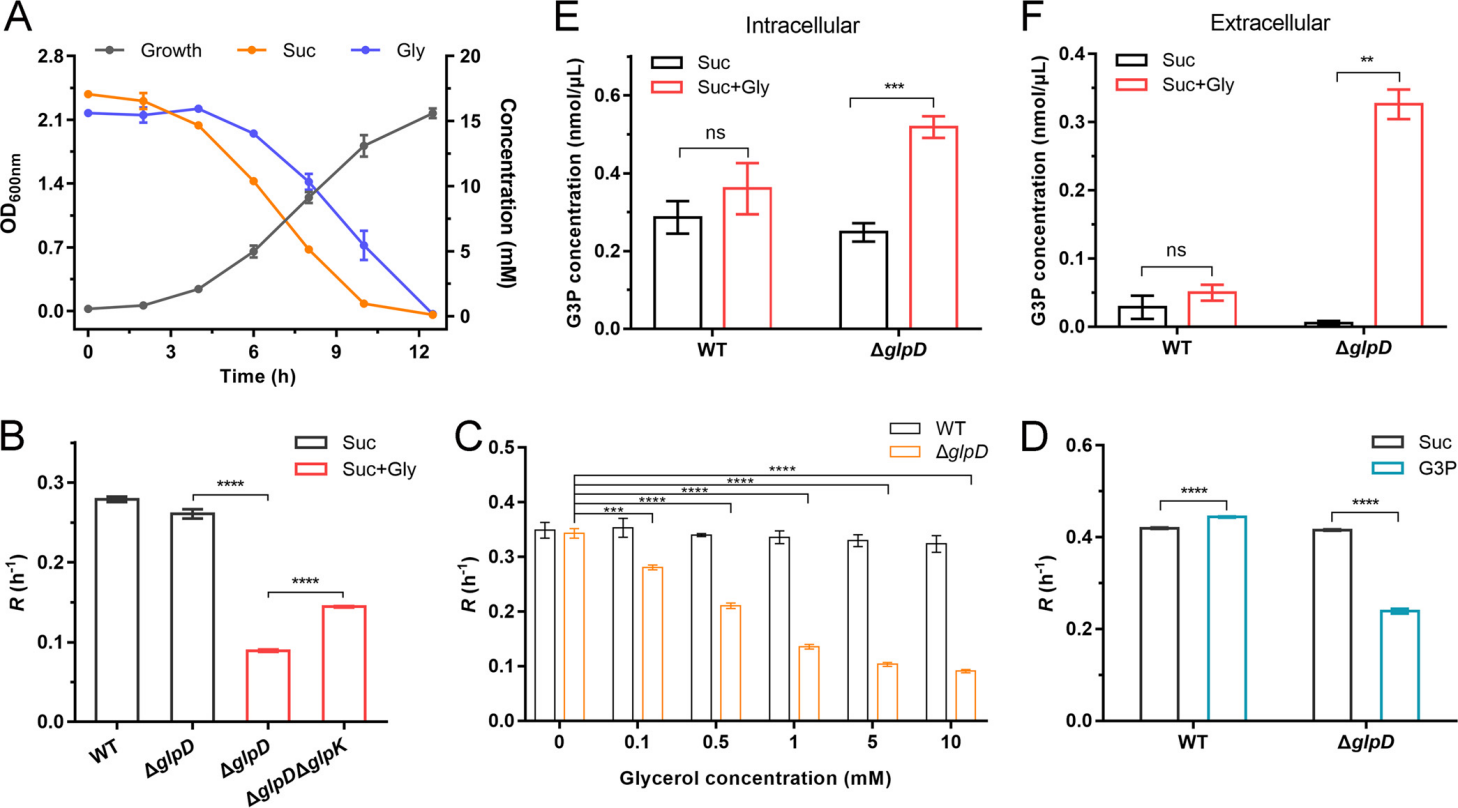
G3P accumulation inhibits the growth of P. aeruginosa PAO1. (A) Growth of P. aeruginosa PAO1 with 20 mM succinate and 20 mM glycerol as the carbon sources. The level of growth (black line) and consumption of succinate (orange line) and glycerol (blue line) were measured. (B) Log-phase growth rate (*R*) of P. aeruginosa PAO1, Δ*glpD*, and Δ*glpD*Δ*glpK* with 20 mM succinate (black columns) or 20 mM succinate and 20 mM glycerol (red columns) as the carbon sources. (C) Log-phase growth rate (*R*) of P. aeruginosa PAO1 (black column) and Δ*glpD* (orange column) with 20 mM succinate and different concentrations of glycerol as the carbon sources. (D) Log-phase growth rate (*R*) of P. aeruginosa PAO1 and Δ*glpD* with 20 mM succinate (black columns) or 20 mM succinate and 10 mM G3P (blue columns) as the carbon sources. (E and F) Intracellular (E) and extracellular (F) G3P concentrations of P. aeruginosa PAO1 and Δ*glpD* grown with 40 mM succinate before and after the addition of 20 mM glycerol. All data shown are the average values of three independent experiments ± SD. Two-tailed *P* values were determined using unpaired *t* tests. **, *P* < 0.01; ***, *P* < 0.001; ****, *P* < 0.0001; ns, no significant difference (*P ≥* 0.05).

P. aeruginosa can cleave the phosphatidylcholine in CF airways, thereby yielding phosphorylcholine, fatty acids, and glycerol (4). In the present study, we observed that P. aeruginosa PAO1 coutilizes glycerol and other favorable carbon sources, such as succinate or glucose (Fig. 3A; Fig. S2A). Δ*glpD* was cultured in MSM containing 20 mM succinate or glucose and 20 mM glycerol. As shown in Fig. 3B; Fig. S2B, exogenous glycerol inhibited the growth of Δ*glpD*, with this inhibitory effect being shown to be dose-dependent (Fig. 3C; Fig. S2C). Exogenous G3P also inhibited the growth of the Δ*glpD* mutant (Fig. 3D). In addition, the knockout of *glpK*, which is responsible for glycerol phosphorylation, was found to partially alleviate the inhibitory effect of glycerol on Δ*glpD* (Fig. 3B; Fig. S2B), thereby indicating a possible correlation between the inhibition of growth caused by a GlpD deficiency and the accumulation of G3P.

10.1128/mbio.02624-22.2 FIG S2 The G3P accumulation inhibits the growth of P. aeruginosa PAO1. (A) Growth of P. aeruginosa PAO1 with 20 mM glucose and 20 mM glycerol as the carbon source. The level of growth (black line) and the consumption of glucose (purple line) and glycerol (blue line) were measured. (B) Log-phase growth rate (*R*) of P. aeruginosa PAO1, Δ*glpD*, and Δ*glpD*Δ*glpK* with 20 mM glucose (black columns) or 20 mM glucose and 20 mM glycerol (red columns) as the carbon sources. (C) Log-phase growth rate (*R*) of P. aeruginosa PAO1 (black column) and Δ*glpD* (orange column) with 20 mM glucose and different concentrations of glycerol as the carbon sources. (D and E) Intracellular (D) and extracellular (E) G3P concentrations of P. aeruginosa PAO1 and Δ*glpD* grown with 40 mM glucose before and after the addition of 20 mM glycerol. All data shown are the average values of three independent experiments ± SD. Two-tailed *P* values were determined using unpaired *t* tests. *, *P* < 0.05; **, *P* < 0.01; ***, *P* < 0.001; ****, *P* < 0.0001; ns, no significant difference (*P* ≥ 0.05). Download FIG S2, TIF file, 0.7 MB. Copyright © 2022 Liu et al. 2022 Liu et al. https://creativecommons.org/licenses/by/4.0/ This content is distributed under the terms of the Creative Commons Attribution 4.0 International license.

The wild-type strain and the Δ*glpD* mutant were cultured to the logarithmic phase of growth in MSM containing 40 mM succinate as the sole carbon source, after which 20 mM glycerol was added and the concentrations of intracellular and extracellular G3P were determined. As shown in Fig. 3E and F, there were significant accumulations of both intracellular and extracellular G3P in Δ*glpD*, whereas we detected no apparent accumulation of G3P in the wild-type strain. Similar results were obtained when Δ*glpD* was cultured in MSM containing glucose as the sole carbon source (Fig. S2D and E).

**FIG 4:**
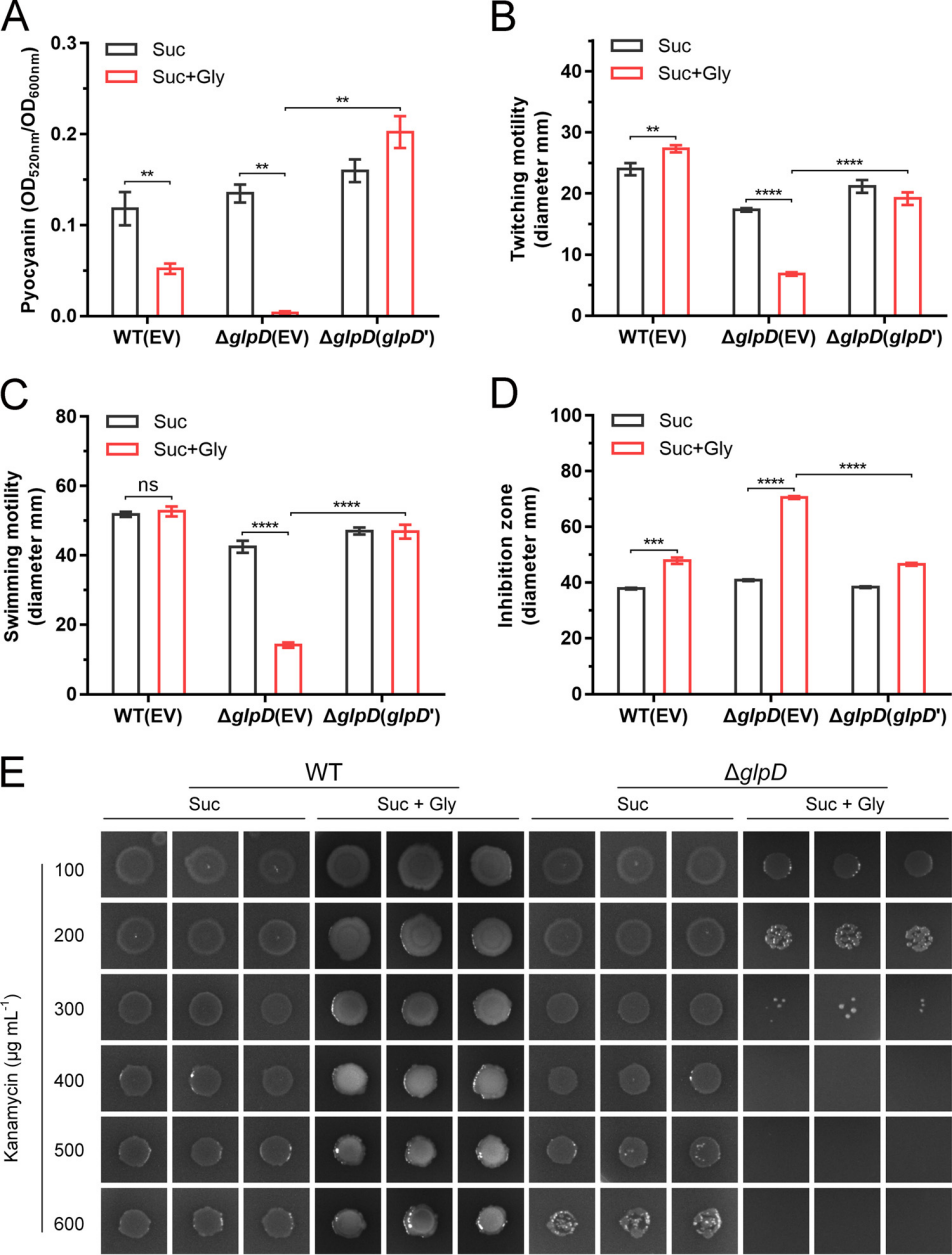
Effects of G3P stress on the pyocyanin production, motilities, oxidative stress tolerance, and antibiotic resistance of P. aeruginosa PAO1. (A to D) Spectrophotometric quantitation of pyocyanin (A). Diameters of subsurface twitching motility zones on 1% agar plates (B). Diameters of surface swimming motility zones on 0.3% agar plates (C). Diameters of bacteriostatic zones generated by 30% (wt/wt) H_2_O_2_ (D). WT(EV), P. aeruginosa PAO1 complemented with the empty vector pBBRMCS-5; Δ*glpD*(EV), Δ*glpD* complemented with the empty vector pBBRMCS-5; Δ*glpD*(*glpD*’), Δ*glpD* complemented with pBBR-*glpD*. (E) Kanamycin sensitivity assay. All strains were grown with 20 mM succinate (Suc) or 20 mM succinate and 20 mM glycerol (Suc + Gly) as the carbon sources. All data shown are the average values of three independent experiments ± SD. Two-tailed *P* values were determined using unpaired *t* tests. **, *P* < 0.01; ***, *P* < 0.001; ****, *P* < 0.0001; ns, no significant difference (*P* ≥ 0.05).

We also examined the pyocyanin production, twitching and swimming motilities, oxidative stress tolerance, and antibiotic resistance of the Δ*glpD* mutant under conditions with exogenous glycerol. As shown in Fig. 4A and Fig. S3A, exogenously supplied glycerol completely prevented the production of pyocyanin by Δ*glpD*. We also observed reductions in the twitching and swimming motilities of Δ*glpD* in the presence of glycerol (Fig. 4B and C; Fig. S3B and C). Exogenous glycerol markedly increased the susceptibility of Δ*glpD* to H_2_O_2_ (Fig. 4D; Fig. S3D) and kanamycin (Fig. 4E). Furthermore, we found that the pyocyanin generation, motilities, and oxidative stress tolerance of the complementary strain Δ*glpD*(*glpD*’) recovered to various degrees (Fig. 4A to D; Fig. S3). Thus, these results indicate that the accumulation of G3P will lead to reductions in the pyocyanin production, motilities, and tolerances of P. aeruginosa PAO1.

10.1128/mbio.02624-22.3 FIG S3 Effects of G3P stress on the pyocyanin production, motilities, and oxidative stress tolerance of P. aeruginosa PAO1. (A to D) Photographs of the acidified extracts of the cultures of P. aeruginosa PAO1 and its derivatives in minimal salt medium (MSM) and stationary-phase with chloroform (A). The pink color is proportional to the amount of pyocyanin. Photographs of subsurface twitching motility zones on 1% agar plates (B). Photographs of surface swimming motility zones on 0.3% agar plates (C). Photographs of bacteriostatic zones generated by 30% (wt/wt) H_2_O_2_ (D). P. aeruginosa PAO1 complemented with empty vector pBBRMCS-5 (EV), Δ*glpD* complemented with empty vector pBBRMCS-5 (EV) and pBBR-*glpD* (*glpD*’) were grown with 20 mM succinate (Suc) or 20 mM succinate and 20 mM glycerol (Suc + Gly) as the carbon sources. All experiments were performed in parallel with at least three biological replicates, and the data shown represent comparable results. Download FIG S3, TIF file, 2.8 MB. Copyright © 2022 Liu et al. 2022 Liu et al. https://creativecommons.org/licenses/by/4.0/ This content is distributed under the terms of the Creative Commons Attribution 4.0 International license.

### Transcriptomic response of P. aeruginosa PAO1 to G3P stress.

We adopted a transcriptomic approach to examine the changes in G3P-induced gene expression in P. aeruginosa PAO1. Having cultured Δ*glpD* in MSM containing 40 mM succinate as the carbon source to an optical density at 600 nm (OD_600nm_) of 1, we added 20 mM glycerol to induce G3P accumulation. Cells treated with exogenous glycerol were subjected to transcriptomic analysis and compared to untreated cells.

**FIG 5:**
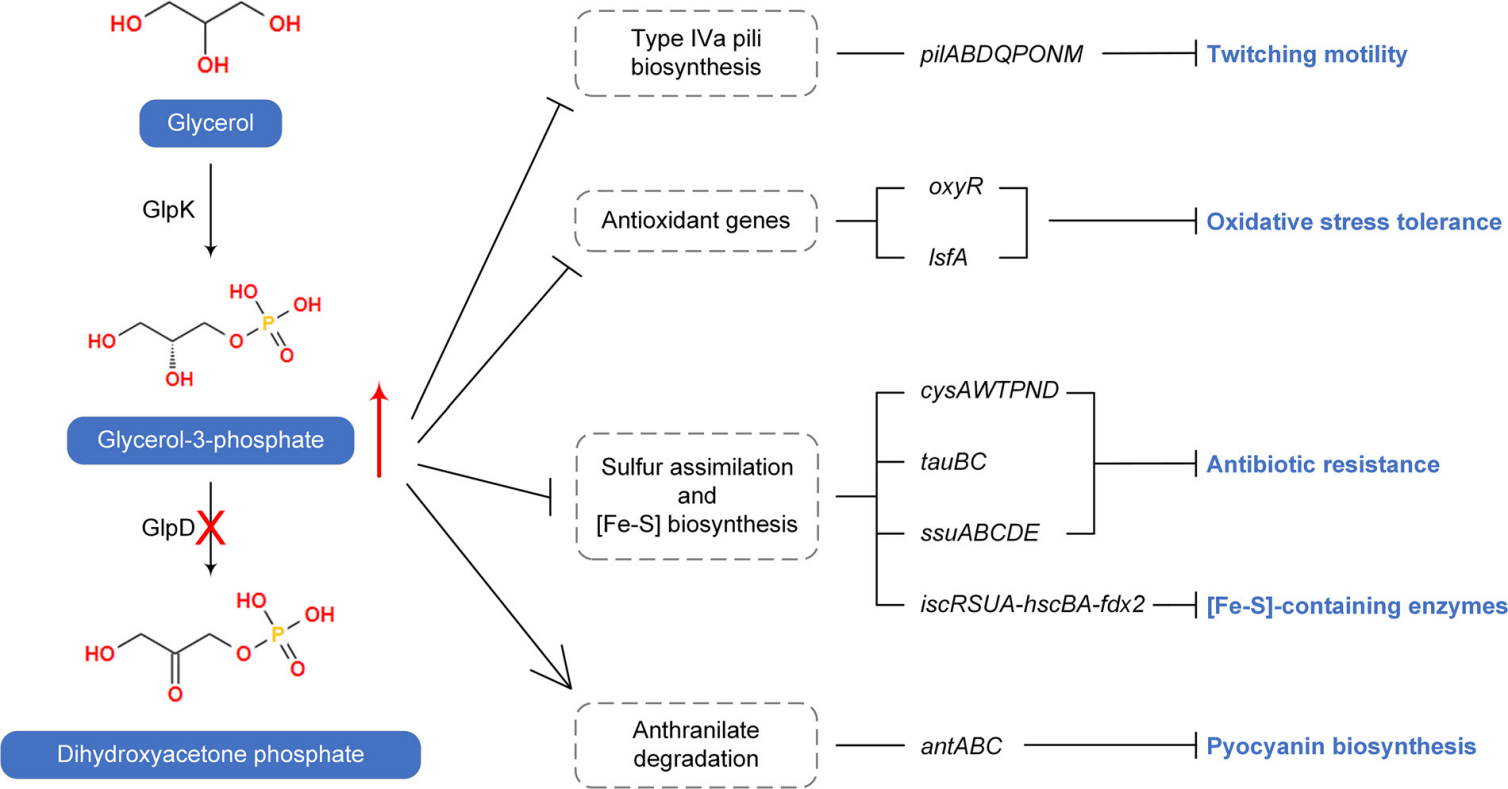
Transcriptomic response of P. aeruginosa PAO1 to G3P stress. The mutation of GlpD blocks glycerol catabolism and results in the accumulation of G3P. Transcriptomic analysis revealed that genes relating to type IVa pili biosynthesis, antioxidants, sulfur assimilation, and [Fe-S] biosynthesis were repressed by G3P accumulation, resulting in reduced twitching motility, oxidative stress tolerance, and antibiotic resistance as well as the impaired functioning of [Fe-S]-containing enzymes. Anthranilate degradation was activated under G3P stress, which may compete with pyocyanin biosynthesis for the common precursor chorismate. Details about the displayed genes can be found in Table S1.

P. aeruginosa is characterized by the production of two major subfamilies of type IV pili, namely, type IVa pili and type IVb pili (29, 30). Type IVa pili are typically responsible for twitching motility, which plays important roles in the adherence of bacteria to surfaces and in the migration of bacteria toward attractants (29). Accordingly, we speculate that a reduction in the expression of genes related to type IVa pili biosynthesis may account for the impaired twitching motility observed in the Δ*glpD* mutant in response to an accumulation of G3P (Fig. 5; Table S1).

10.1128/mbio.02624-22.7 TABLE S1 Fold changes of mRNA transcripts in P. aeruginosa PAO1 (Δ*glpD*) under G3P stress. Download Table S1, DOC file, 1.3 MB. Copyright © 2022 Liu et al. 2022 Liu et al. https://creativecommons.org/licenses/by/4.0/ This content is distributed under the terms of the Creative Commons Attribution 4.0 International license.

OxyR positively regulates a series of defensive genes against oxidative stress (31), and 1-cys peroxiredoxin LsfA is similarly involved in protecting cells from superoxide-induced stress (32). Thus, it is conceivable that the significantly reduced levels of *oxyR* and *lsfA* mRNA observed in response to an accumulation of G3P may account for the increased susceptibility of P. aeruginosa to oxidative stress under these conditions (Fig. 5; Table S1). In bacteria, sulfonates and sulfate esters are considered to serve as the major sources of sulfur for cysteine and methionine biosynthesis (33). In P. aeruginosa, the H_2_S generated from cysteine is required for defense against antibiotics (34). Most genes associated with sulfate transport (*cysAWTP*) and metabolism (*cysND*), taurine transport (*tauBC*), and alkanesulfonate metabolism (*ssuABCDE*) have been shown to be downregulated (33) (Table S1), indicating that the attenuated assimilation of sulfur might affect the resistance of P. aeruginosa to antibiotics under conditions of G3P stress (Fig. 5). Similarly, the genes associated with iron-sulfur cluster ([Fe-S]) biosynthesis (*iscRSUA-hscBA-fdx2*) were also significantly downregulated in the Δ*glpD* mutant in response to the addition of glycerol (Table S1), which may influence numerous physiological processes involving [Fe-S]-containing enzymes (35) (Fig. 5).

Notably, however, we found that the genes involved in the synthesis of pyocyanin (*phz* operon) were upregulated (Table S1), which appears to be inconsistent with the observed reduction in pyocyanin production in cells subjected to G3P stress (Fig. 4A). Chorismate is the precursor of pyocyanin biosynthesis catalyzed by the enzymes encoded by the *phz* operon (36). It can also be converted to anthranilate and is thereafter degraded to catechol by *antABC*-encoded anthranilate dioxygenase before it eventually enters the tricarboxylic acid (TCA) cycle (Fig. S4). The transcriptomic analysis revealed that *antABC* was more highly upregulated than was the *phz* operon (Table S1), which may thus divert the flux of chorismate into anthranilate degradation and thereby result in a reduction in pyocyanin production (Fig. 5).

10.1128/mbio.02624-22.4 FIG S4 Transcriptional responses of genes related to chorismate metabolism to G3P stress in P. aeruginosa PAO1. Genes that are significantly upregulated are labeled in red. Phz, enzymes in pyocyanin biosynthesis pathway, including PhzA1-G1, PhzA2-G2, PhzM, and PhzS. Download FIG S4, TIF file, 0.3 MB. Copyright © 2022 Liu et al. 2022 Liu et al. https://creativecommons.org/licenses/by/4.0/ This content is distributed under the terms of the Creative Commons Attribution 4.0 International license.

### ATP generation is reduced in P. aeruginosa PAO1 under G3P stress.

**FIG 6:**
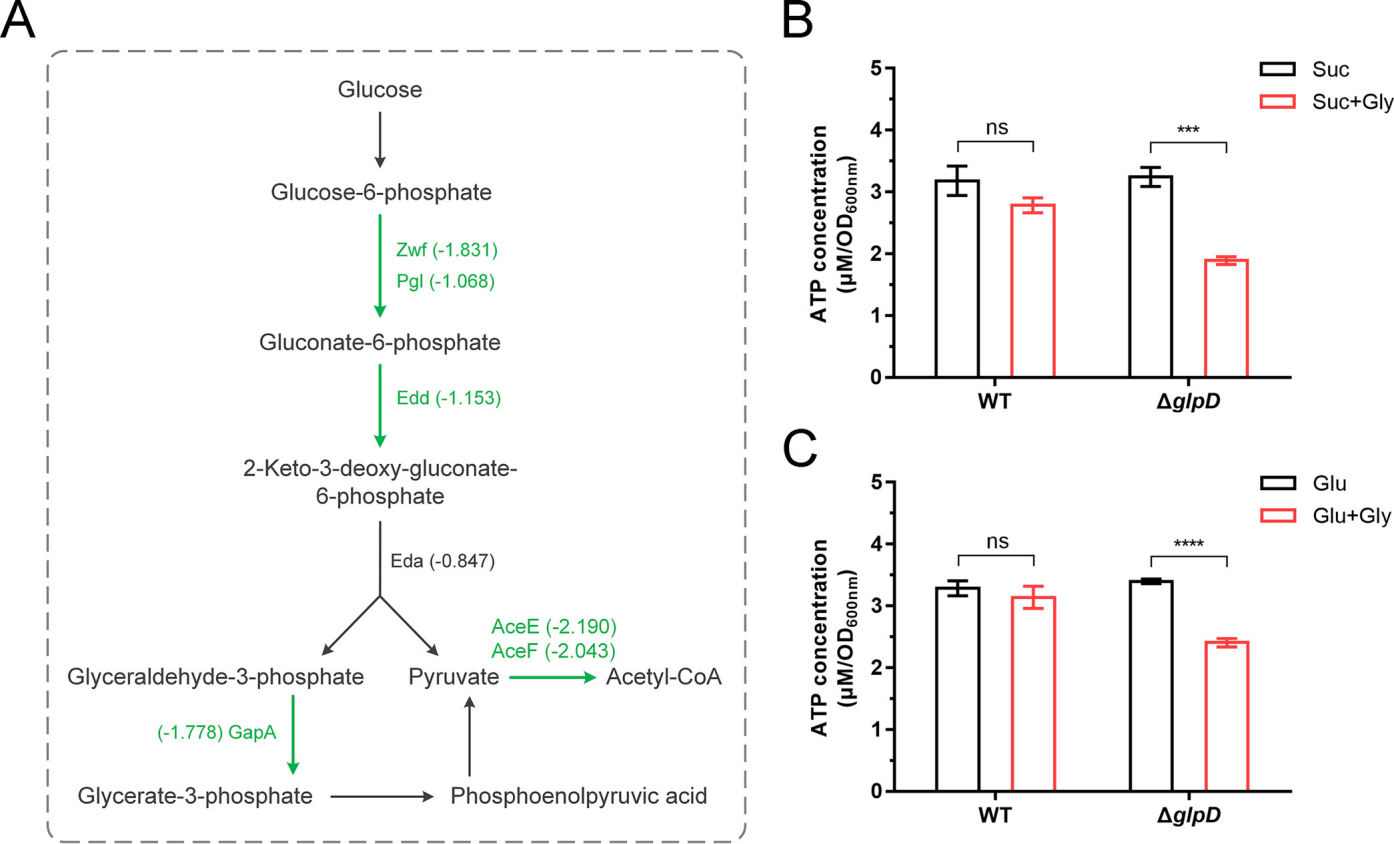
G3P accumulation inhibits the expression of genes in the Entner-Doudoroff (ED) pathway and adenosine triphosphate (ATP) production of P. aeruginosa PAO1. (A) Transcriptional response of genes in the ED pathway to G3P stress in P. aeruginosa PAO1. Genes that are significantly downregulated are labeled in green. (B and C) ATP detection of P. aeruginosa PAO1 and Δ*glpD* grown with 40 mM succinate (B) or 40 mM glucose (C) before and after the addition of 20 mM glycerol. All data shown are the average values of three independent experiments ± SD. Two-tailed *P* values were determined using unpaired *t* tests. ***, *P* < 0.001; ****, *P* < 0.0001; ns, no significant difference (*P* ≥ 0.05).

P. aeruginosa employs the ED pathway for glycolysis (37), the participating genes of which are arranged in two regulons. One of these regulons consists of *zwf* (encoding glucose-6-phosphate 1-dehydrogenase), *pgl* (encoding 6-phosphogluconolactonase), and *eda* (encoding 2-keto-3-deoxy-6-phosphogluconate aldolase), whereas the other consists of *edd* (encoding 6-phosphogluconate dehydratase) and *gapA* (encoding glyceraldehyde-3-phosphate dehydrogenase). Transcriptome sequencing results revealed a clear downregulation of these ED pathway regulons in Δ*glpD* in response to G3P accumulation (Fig. 6A; Table S1).

Pyruvate dehydrogenase catalyzes the oxidative decarboxylation of pyruvate to acetyl-CoA (Fig. 6A). In the present study, we detected a downregulation of the expression of the pyruvate dehydrogenase complex genes *aceE* and *aceF* (38) in Δ*glpD* following the addition of glycerol (Table S1). We also detected a reduction in the transcription of *bo*_3_ quinol oxidase (encoded by *cyoABCDE*) of the electron transport chain under aerobic respiration (39), whereas the transcription of multiple genes involved in nitrate-dependent anaerobic respiration (*narK1K2GHJI*, *nirSMCFDLGHJEN*, and *nosRZDFYL*) (40) was increased under G3P accumulation (Table S1). These findings indicate that G3P accumulation has an inhibitory effect on aerobic respiration. This G3P-induced downregulation of glucose metabolism in aerobic respiration would presumably result in a reduced ATP pool. Thus, we also assayed the levels of ATP in P. aeruginosa PAO1 and Δ*glpD* before and after the addition of glycerol. As expected, we detected a reduction in ATP levels in Δ*glpD* under G3P accumulation (Fig. 6B and C).

### Identification of putative G3PPs in P. aeruginosa PAO1.

**FIG 7:**
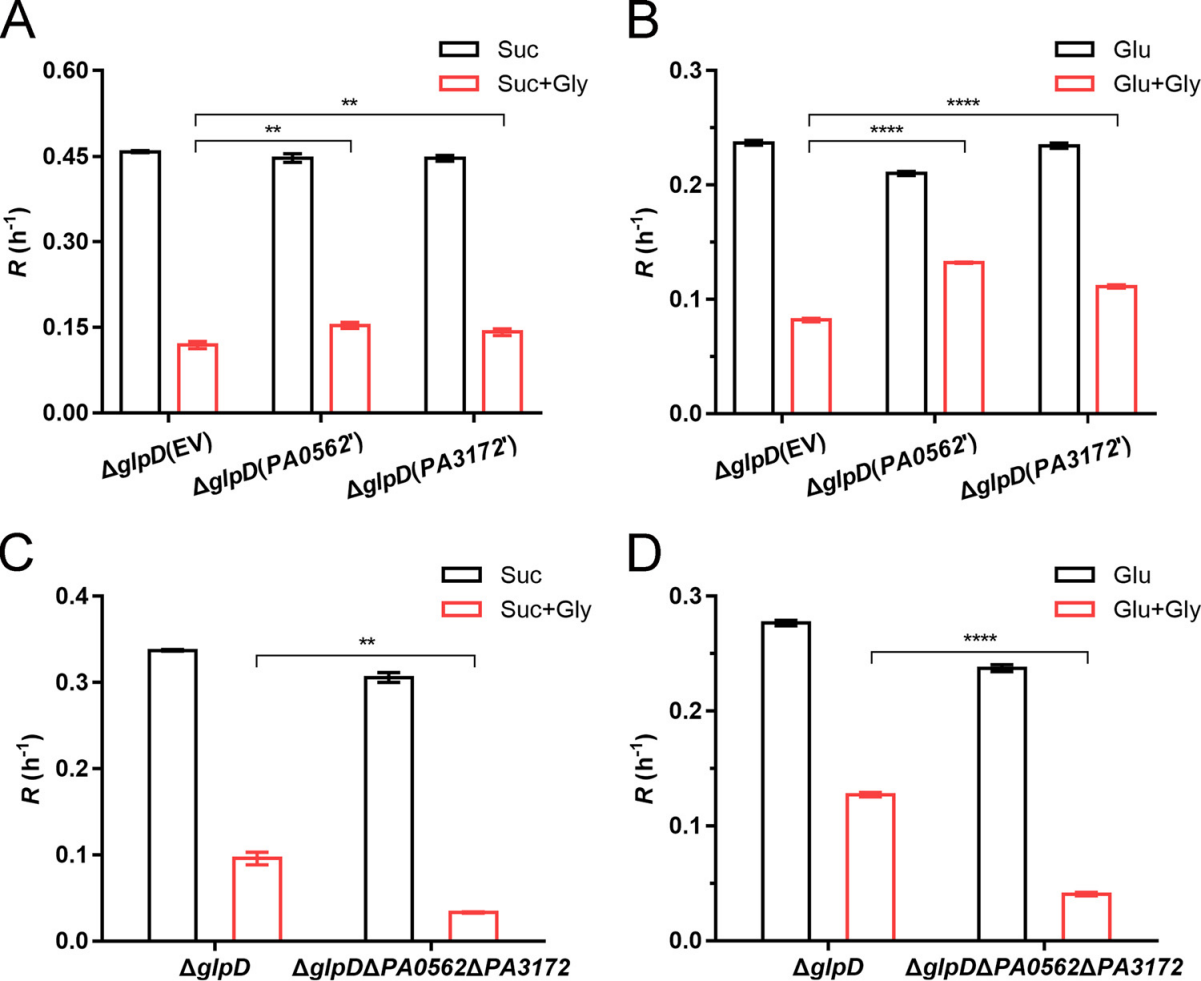
PA0562 and PA3172 contribute to G3P stress alleviation. (A and B) Log-phase growth rate (*R*) of Δ*glpD* complemented with empty vector pBBRMCS-5 (EV), pBBR-*PA0562* (*PA0562*’), or pBBR-*PA3172* (*PA3172*’). (C and D) Log-phase growth rate (*R*) of Δ*glpD* and Δ*glpD*Δ*PA0562*Δ*PA3172*. All strains were grown with either 20 mM succinate (A and C) or 20 mM glucose (B and D) as the carbon source. The addition of 5 mM glycerol is indicated by the red columns. All data shown are the average values of three independent experiments ± SD. Two-tailed *P* values were determined using unpaired *t* tests. **, *P* < 0.01; ****, *P* < 0.0001.

Phosphatases that convert G3P into glycerol have been identified in many organisms (10, 11). We performed a position-specific-iterated BLAST search using the amino acid sequence of S. cerevisiae G3PP RHR2 (NP_012211.2) as a query. Three homologous proteins belonging to the haloacid dehalogenase (HAD)-like hydrolases were found in P. aeruginosa PAO1, including PA0562, PA3172, and PA2067, which share 20%, 23%, and 22% identities with S. cerevisiae G3PP RHR2, respectively (Fig. S5). According to the annotations in the National Center for Biotechnology Information (NCBI) database, PA0562 and PA3172 may have phosphatase activities, while PA2067 may be a *β*-phosphoglucomutase YcjU. By overexpressing these three candidate G3PPs in Δ*glpD*, we found that only overexpressed PA0562 and PA3172 alleviated the effects of G3P stress and resulted in an increase in the log-phase growth rate of Δ*glpD* (Fig. 7A and B; Fig. S6). Consistently, the knockout of *PA0562* and *PA3172* in Δ*glpD* had the effect of enhancing the inhibition of growth attributable to accumulated G3P (Fig. 7C and D). These findings imply that PA0562 and PA3172 are G3PPs of P. aeruginosa PAO1.

10.1128/mbio.02624-22.5 FIG S5 Sequence alignment of putative G3PPs in P. aeruginosa PAO1 and S. cerevisiae RHR2. The alignment was performed using BioEdit software. Sequence conservation is indicated as follows: (*), fully conserved amino acids; (:), strongly conserved amino acids; (.), weakly conserved amino acids. Download FIG S5, TIF file, 1.6 MB. Copyright © 2022 Liu et al. 2022 Liu et al. https://creativecommons.org/licenses/by/4.0/ This content is distributed under the terms of the Creative Commons Attribution 4.0 International license.

10.1128/mbio.02624-22.6 FIG S6 PA2067 is not responsible for G3P stress alleviation. (A and B) Log-phase growth rate (*R*) of Δ*glpD* complemented with empty vector pBBRMCS-5 (EV) and pBBR-*PA2067* (*PA2067*’) with 20 mM succinate (A) or glucose (B) as the carbon source. The addition of 5 mM glycerol is indicated by the red columns. All data shown are the average values of three independent experiments ± SD. Two-tailed *P* values were determined using unpaired *t* tests; ns, no significant difference (*P* ≥ 0.05). Download FIG S6, TIF file, 0.2 MB. Copyright © 2022 Liu et al. 2022 Liu et al. https://creativecommons.org/licenses/by/4.0/ This content is distributed under the terms of the Creative Commons Attribution 4.0 International license.

**FIG 8:**
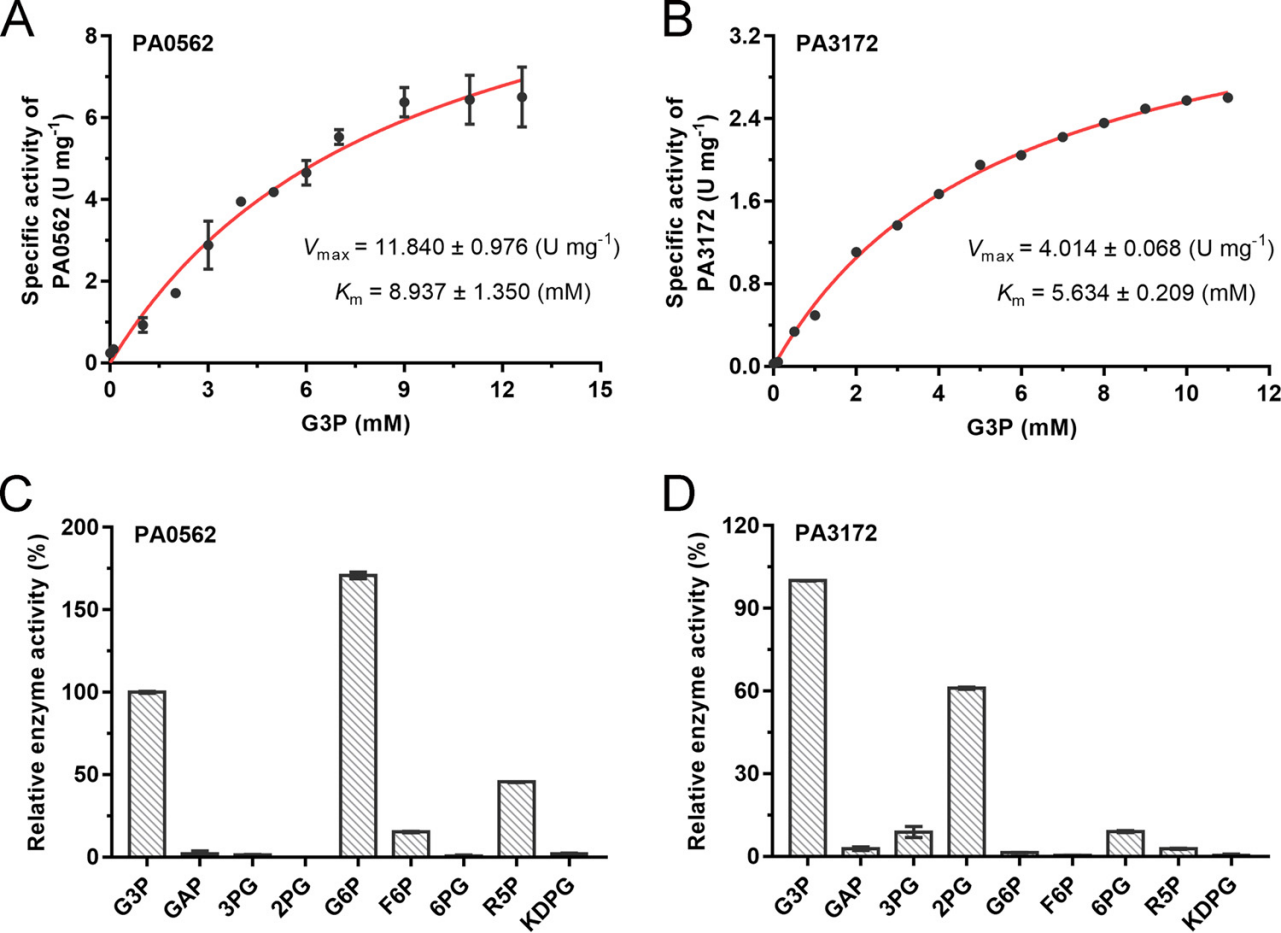
Enzymatic characterization of PA0562 and PA3172 in P. aeruginosa PAO1. (A and B) The kinetic parameters of PA0562 (A) and PA3172 (B) were deduced from Michaelis-Menten regression curves. (C and D) Relative activities of PA0562 (C) and PA3172 (D) toward different substrates at 10 mM. The specific activity toward G3P was defined as 100%. GAP, 3-phosphoglyceraldehyde; 3PG, 3-phosphoglycerate; 2PG, 2-phosphoglycerate; G6P, glucose-6-phosphate; F6P, fructose-6-phosphate; 6PG, 6-phosphogluconate; R5P, ribose-5-phosphate; KDPG, 2-keto-3-deoxy-6-phosphogluconate. All data shown are the average values of three independent experiments ± SD.

Then, the enzymes PA0562 and PA3172 were overexpressed in E. coli BL21(DE3) and purified through nickel affinity chromatography. The kinetic parameters of PA0562 and PA3172 toward G3P were determined (Fig. 8A and B). The apparent *K*_m_ values of PA0562 and PA3172 toward G3P were 8.937 ± 1.350 mM and 5.634 ± 0.209 mM, respectively. The corresponding *V*_max_ values were estimated to be 11.840 ± 0.976 U·mg^−1^ and 4.014 ± 0.068 U·mg^−1^, respectively.

PA0562 and PA3172 belong to the HAD-like phosphatases (41). The members of these phosphatases in E. coli (42) and P. fluorescens (43) have been demonstrated to have broad substrate spectra. In contrast, it has been found that G3PPs from glycerol-overproducing microbes, such as S. cerevisiae (44) and C. glutamicum (11), exhibit high substrate specificity and catalytic efficiency. From the substrate profiles of PA0562 and PA3172, we established that, in addition to G3P, PA0562 can catalyze the dephosphorylation of glucose-6-phosphate, ribose-5-phosphate, and fructose-6-phosphate, with the highest activity being detected when glucose-6-phosphate was used as a substrate (Fig. 8C). PA3172 also had a relatively broad substrate spectrum, with its activities toward 2-phosphoglycerate, 3-phosphoglycerate, and 6-phosphogluconate all being lower than its activity toward G3P (Fig. 8D).

## DISCUSSION

P. aeruginosa is a major human nosocomial pathogen that causes fatal infections in patients with CF and immune deficiencies (2). However, treating infections attributable to P. aeruginosa is becoming increasingly difficult due to the widespread development of antibiotic resistance (2). Glycerol catabolism has been implicated in the adaptation of P. aeruginosa to the CF lung environment (4). In the present study, we identified the functions of glycerol metabolic genes, including *glpF*, *glpK*, *glpR*, and *glpD*, in P. aeruginosa PAO1. Notably, we established that, rather than glycerol, the glycerol catabolism regulator GlpR in P. aeruginosa PAO1 uses G3P as its effector (Fig. S1D and E). We also investigated the synthesis, degradation, and transport mechanisms of G3P in P. aeruginosa PAO1. It was found that G3P is involved in maintaining the cellular homeostasis of P. aeruginosa PAO1 and that an accumulation of G3P leads to reductions in the growth of cells (Fig. 3; Fig. S2), production of several virulence factors, tolerance to oxidative stress, and resistance to kanamycin (Fig. 4; Fig. S3).

Although the specific mechanism by which the accumulation of G3P induces phenotypic alterations in P. aeruginosa PAO1 remains to be ascertained, we suspect that the growth inhibition caused by G3P stress may be associated with a remodeling of respiration and a reduction in ATP generation (Fig. 6). Furthermore, the observed decline in the twitching motility of P. aeruginosa PAO1 exposed to G3P stress (Fig. 4B) could be attributable to the repressed expression of type IVa pili biosynthesis genes (Table S1). A transcriptomic analysis further indicated that sulfur metabolism and antioxidant gene expression may also be influenced by G3P accumulation (Table S1), eventually resulting in a reduced tolerance to oxidative stress and a reduced resistance to kanamycin (Fig. 4D and E). Shuman et al. previously observed the upregulated expression of the *phz* operons in late-logarithmic-phase cultures of Δ*glpD* (45); however, a reduction in the production of pyocyanin was also reported in the Δ*glpD* mutants of P. aeruginosa strains FRD1 and PAO1 (46), which is concordant with our findings in the present study (Fig. 2A and Fig. 4A). We suspect that this reduction in pyocyanin production might be associated with a diversion of the metabolic flux of the key precursor, chorismate, to anthranilate degradation mediated via an upregulated expression of *antABC* (Fig. S4; Table S1). Pyocyanin is secreted in the stationary-phase of P. aeruginosa growth (47), and the downregulated *phz* in stationary-phase cells may also account for the reduced production of pyocyanin in the *glpD* mutant of P. aeruginosa (45).

The cellular toxicity caused by the accumulation of G3P appears to be a phenomenon shared by both prokaryotes and eukaryotes, as the negative effects of excess G3P on growth have been observed in E. coli (9, 48), S. cerevisiae (10), C. glutamicum (11), M. tuberculosis (49), and C. elegans (50). Moreover, HAD-like G3PPs that dephosphorylate G3P to glycerol and prevent an accumulation of G3P have been identified in S. cerevisiae (10), C. glutamicum (11), and C. elegans (50). In the present study, we identified and characterized the phosphatases PA0562 and PA3172 in P. aeruginosa PAO1, and the overexpression of PA0562 and PA3172 was found to alleviate G3P-induced growth inhibition, whereas the knockout of these two phosphatases potentiated the inhibitory effects of G3P (Fig. 7). G3PPs from glycerol-producing strains, such as S. cerevisiae (44) and C. glutamicum (11), exhibit high substrate specificity, which may be attributable to these microbes often having to contend with high G3P flux pressure. In contrast, we found that PA0562 and PA3172, like the HAD-like phosphatases in E. coli and P. fluorescens (42, 43), are characterized by relatively broad substrate spectra (Fig. 8C and D). The relaxed specificities of PA0562 and PA3172 imply that these phosphatases may play roles in other metabolic pathways or in the detoxification of other phosphorylated metabolites. For example, PA3172 has been found to catalyze the dephosphorylation of *N*-acetylmuramic acid-6-phosphate to *N*-acetylmuramic acid in the peptidoglycan recycling pathway (51).

Antibiotics are indispensable in the control of infections by pathogens. However, the overuse of antibiotics accelerates the evolution of multidrug-resistant bacteria (12). Accordingly, there is a constant need to identify novel drug targets in bacterial metabolic pathways and present new opportunities for the control of drug-resistant bacteria (52). For example, the catabolism of fatty acids is essential for the *in vivo* persistence of M. tuberculosis. It has been demonstrated in a mouse infection model that the deletion of malate synthase, a key enzyme in the glyoxylate shunt pathway, reduces the infectivity of M. tuberculosis (16). The susceptibility of the malate synthase mutant of M. tuberculosis may be linked to the accumulation of glyoxylate, a toxic metabolite produced during the catabolism of fatty acids (16). Based on its crystal structure and its catalytic mechanism in M. tuberculosis, potent inhibitors of malate synthase have been developed for tuberculosis therapeutics (53). With respect to P. aeruginosa, it has been established that the adaptive evolution of P. aeruginosa in the host lung environment is associated with the metabolism of glycerol (4, 46). In the present study, we found that G3P stress that is induced by the deletion of GlpD reduces the growth and virulence factor production of P. aeruginosa PAO1 (Fig. 3 and Fig. 4). Thus, we believe that GlpD may also have potential therapeutic utility as a target for the control of P. aeruginosa infections. The structure of GlpD in E. coli has been resolved (54), and the GlpD in P. aeruginosa PAO1 displays 59% identity with the E. coli homolog and possesses consistent active sites. Thus, the inhibitors of GlpD could be designed and developed for the control of infections attributable to P. aeruginosa.

Bacterial antibiotic resistance has been shown to be closely correlated with physiological metabolism (13–15). In this context, metabolic modulations that enhance antibiotic efficiency have been used to manage infections induced by antibiotic-resistant bacteria. For example, exogenous glucose or alanine can increase the TCA flux, resulting in the stimulation of kanamycin uptake, which has been demonstrated to kill multidrug-resistant bacteria both *in vitro* and in a mouse model of a urinary tract infection (55). Furthermore, it has been demonstrated that exposure to exogenous glutamine can increase the membrane permeability of many pathogens and can thereby promote the uptake of ampicillin to restore its antimicrobial activity (14). In the present study, we established that glycerol can greatly reduce the bactericidal concentration of kanamycin against Δ*glpD* (Fig. 4E) and that the reduced resistance phenotype is associated with a perturbation of G3P metabolism. Consequently, a GlpD inhibitor that influences G3P metabolism could potentially be administered in combination with antibiotics for a more effective control of difficult-to-treat P. aeruginosa infections.

In summary, we ascertained the functions of genes involved in G3P metabolism and demonstrated the importance of G3P homeostasis in P. aeruginosa PAO1. The accumulation of G3P reduced the virulence factor generation, inhibited the growth, and reduced the tolerance of P. aeruginosa PAO1 to oxidative and antibiotic stresses. Although further studies using infection models will be necessary to confirm whether GlpD plays an essential role *in vivo*, based on its involvement in the virulence phenotype, our findings provide evidence to indicate its potential utility as a drug target for the treatment of P. aeruginosa infections.

## MATERIALS AND METHODS

### Bacterial strains and culture conditions.

The bacterial strains and plasmids used in this study are listed in Table S2. P. aeruginosa PAO1 and its derivatives were cultured in MSM (56) with different carbon sources at 180 rpm and 37°C. E. coli strains DH5α and BL21(DE3) were cultured in LB medium at 180 rpm and 37°C. When necessary, antibiotics were added to the medium at the following concentrations, unless otherwise indicated: ampicillin, 100 μg mL^−1^; gentamicin, 30 μg mL^−1^; tetracycline, 40 μg mL^−1^, respectively.

10.1128/mbio.02624-22.8 TABLE S2 Strains and plasmids used in this study. Download Table S2, DOC file, 1.3 MB. Copyright © 2022 Liu et al. 2022 Liu et al. https://creativecommons.org/licenses/by/4.0/ This content is distributed under the terms of the Creative Commons Attribution 4.0 International license.

### Gene knockout and complementation.

The plasmids and primers used for the gene knockout and complementation are listed in Table S3. Mutants of P. aeruginosa PAO1 were constructed through the suicide plasmid pK18*mobsacB*-*tet*-mediated allelic exchange (57, 58). For the complementation of *PA0562*, the full-length of *PA0562* was amplified from the genomic DNA of P. aeruginosa PAO1 using primers *PA0562*-F/*PA0562*-R, and the PCR product was cloned into the vector pBBR1MCS-5 (59) to get plasmid pBBR-*PA0562*. The recombinant plasmid pBBR-*PA0562* was transformed into Δ*glpD* via electroporation. The correct transformants were selected on LB plates containing 45 μg mL^−1^ gentamicin. Δ*glpD* complemented with pBBR-*PA2067*, pBBR-*PA3172*, and pBBR-*glpD* were obtained by using the same procedure.

10.1128/mbio.02624-22.9 TABLE S3 Primers used in this study. Download Table S3, DOC file, 1.3 MB. Copyright © 2022 Liu et al. 2022 Liu et al. https://creativecommons.org/licenses/by/4.0/ This content is distributed under the terms of the Creative Commons Attribution 4.0 International license.

### Modeling growth.

The growth of P. aeruginosa PAO1 and its derivatives in MSM with different carbon sources were monitored by using a BioScreen microbiology reader (BioScreen C Labsystems, Helsinki, Finland). The OD_600nm_ value of zero and different time points were defined as A_0_ and A, respectively. Growth curves obtained by plotting the natural logarithm of A/A_0_ versus time were fitted using the Gompertz (3 parameter) model (60). The log-phase growth rate *R* (h^−1^) was calculated as the slope of the linear fit between the exponential-phase natural logarithm of A/A_0_ and the time.

### Expression and purification of proteins.

The *glpR* gene was amplified from the genomic DNA of P. aeruginosa PAO1 using primers GlpR-F/GlpR-R and was cloned into the vector pETDuet-1. The recombinant plasmid pETDuet-*glpR* was introduced into E. coli BL21(DE3). The constructed strain was then grown to an OD_600nm_ of 0.6 to 0.8 in LB medium at 37°C and 180 rpm. Then, 1 mM isopropyl-*β*-d-thiogalactopyranoside (IPTG) was added to induce protein expression at 16°C and 160 rpm for 12 h. Cells were harvested and suspended in buffer A (50 mM Tris-HCl, 20 mM imidazole, and 500 mM NaCl, pH 7.4) containing 1 mM phenylmethanesulphonyl fluoride (PMSF) and 10% (vol/vol) glycerol. Then, the cells were lysed via sonication on ice, and the lysate was centrifuged at 12,000 × *g* for 40 min at 4°C. The supernatant was loaded onto a HisTrap HP column (5 mL) equilibrated with buffer A and eluted with buffer B (50 mM Tris-HCl, 1 M imidazole, and 500 mM NaCl, pH 7.4). The purified protein was analyzed by 13% sodium dodecyl sulfate-polyacrylamide gel electrophoresis (SDS-PAGE) and was quantified using a Bradford protein assay kit (Sangon, China). PA0562 and PA3172 were expressed and purified by using the same procedure.

### Electrophoretic mobility shift assay (EMSA).

The DNA fragments of the *glpD* promoter, *glpFK* promoter, and control probe were amplified from the genomic DNA of P. aeruginosa PAO1 using primers P*_glpD_*-F/P*_glpD_*-R, P*_glpFK_*-F/P*_glpFK_*-R, and CP-F/CP-R (Table S3), respectively. The 20 μL reaction solution contained 3.5 nM target DNA fragments and control probe, 10 mM *sn*-glycerol 3-phosphate lithium salt (Sigma-Aldrich) or glycerol, and 500 nM purified GlpR in the binding buffer (pH 7.4, 10 mM Tris-HCl, 50 mM KCl, 0.5 mM EDTA, 10% [vol/vol] glycerol, 1 mM dithiothreitol [DTT]). The mixed solutions were incubated at 30°C for 30 min and loaded on 6% native polyacrylamide gels. Electrophoresis was performed at 170 V in 1× TBE buffer (pH 8.3) for 45 min. The gels were stained with SYBR green I (TaKaRa, China) for 20 min and then photographed using a G:BOX F3 gel documentation system (Syngene, USA).

### Quantification of pyocyanin.

Pyocyanin produced by P. aeruginosa PAO1 and its derivatives was quantified as previously described (61). Stationary-phase bacterial cultures (1.5 mL) were centrifuged at 13,000 × *g* for 5 min. Then, 1.25 mL of the supernatant was extracted with 0.75 mL chloroform. After centrifuging at 13,000 × *g* for 1 min, the upper water layer was removed, and the chloroform layer was mixed with 0.25 mL 0.2 M HCl and centrifuged again. Finally, 0.2 mL of the upper aqueous layer was transferred to a 96-well plate with a clear bottom, and the absorbance at 520 nm was measured by SpectraMax Plus 384 plate reader (Molecular Devices, USA). The final absorbance was normalized to the cell densities (OD_600nm_).

### Motility assays.

For the twitching motilities of P. aeruginosa PAO1 and its derivatives, a single colony was stab inoculated to the bottom of LB or MSM plates containing 1% Difco bacto-agar by using a sterile toothpick. For the swimming motilities of P. aeruginosa PAO1 and its derivatives, a single colony was inoculated on the surface of MSM plates containing 0.3% Difco bacto-agar. After incubation at 37°C for 24 h, the zones of motility were measured and photographed using a G:BOX F3 gel documentation system (Syngene, USA).

### Congo red binding assay.

P. aeruginosa PAO1 and its derivatives were cultured overnight in LB and adjusted to an OD_600nm_ of 1 with normal saline. Then, 2 μL of the bacterial suspension was inoculated on the surface of LB agar plates containing 40 μg mL^−1^ Congo red. The plates were photographed after incubation at 25°C for 4 days.

### H_2_O_2_ susceptibility measurement.

The overnight cultures of P. aeruginosa PAO1 and its derivatives were adjusted to an OD_600nm_ of 0.035 with normal saline and were inoculated into 50 mL of melted LB or MSM agar (1.6%) with medium cooling to the appropriate temperature. After solidification, sterile filter paper disks (approximately 6 mm) were placed in the center of the plate, and 10 μL of a 30% (wt/wt) H_2_O_2_ solution was instilled. After incubation at 37°C for 36 h, the zones of inhibition were measured and photographed using a G:BOX F3 gel documentation system (Syngene, USA).

### Antibiotic resistance test.

MSM agar (1.8%) plates with 20 mM succinate and increased concentrations of kanamycin were prepared and dried overnight. When necessary, 20 mM glycerol was also added to the MSM agar to induce G3P accumulation. The overnight cultures of P. aeruginosa PAO1 and its derivatives were washed and adjusted to an OD_600nm_ of 0.05 with normal saline. Then, 5 μL of the bacterial suspension was inoculated on the plates and cultured at 37°C for 4 days. The plates were photographed using a G:BOX F3 gel doc system (Syngene, USA).

### Quantification of G3P.

The concentration of G3P was determined by a commercial Glycerol 3-phosphate Colorimetric Assay Kit (MAK207, Sigma-Aldrich), following the manufacturer’s instructions. P. aeruginosa PAO1 and Δ*glpD* were grown to an OD_600nm_ value of 1 in MSM containing 40 mM succinate or glucose as the carbon source. Then, 20 mM glycerol was added, and the strains were cultivated for 1.5 h. Bacterial cultures sampled before and after the glycerol treatment were subjected to G3P quantification. For the extracellular G3P assays, 15 μL of the culture medium supernatant was used for measurement. For the intracellular G3P assays, the cells of P. aeruginosa PAO1 and Δ*glpD* were centrifuged, washed, and adjusted to an OD_600nm_ of 7.5 in 200 μL of G3P assay buffer for cell lysis. After centrifugation at 13,000 × *g* for 5 min, 10 μL of the centrifugal supernatant was used for measurement. The content of G3P was calculated based on the standard curve and was normalized by the sample volume added to the detection system.

### Transcriptional profiling and analysis.

Δ*glpD* was cultured to an OD_600nm_ value of 1 in MSM containing 40 mM succinate as the carbon source. Then, 20 mM glycerol was added, and the strain was cultivated for 1.5 h. Cells of Δ*glpD* sampled before and after the glycerol treatment were quick-frozen in liquid nitrogen. Transcriptional profiling was performed by Shanghai Biozeron Biotechnology Co., Ltd. (China). Total RNA was extracted using the TRIzol Reagent (Invitrogen). RNA-seq strand-specific libraries were prepared following the TruSeq RNA sample preparation kit from Illumina (San Diego, CA). The libraries were sequenced using an Illumina HiSeq 2000. The clean reads were aligned to the reference genome of P. aeruginosa PAO1 using the Rockhopper software package. The differential expression analysis was conducted using edgeR.

### Detection of ATP.

The intracellular ATP of P. aeruginosa PAO1 and Δ*glpD* was assessed by using the BacTiter-Glo Microbial Cell Viability Assay Kit (Promega), following the manufacturer’s instructions. The sampling process was similar to that described in the G3P quantification methods. The bacterial cultures were centrifuged, washed, and resuspended to an OD_600nm_ of about 0.3. Subsequently, 100 μL of the suspension was transferred into a black 96-well plate, mixed with an equal volume of the BacTiter-Glo Reagent, and incubated for 5 min at room temperature in the dark. The luminescence was detected by using an EnSight microplate reader (PerkinElmer, USA). The concentration of ATP was calculated based on the standard curve and was normalized by OD_600nm_.

### Enzymatic assay of G3PPs.

The activities of PA0562 and PA3172 were assayed in 1 mL of reaction solution containing 0.2 mg mL^−1^ of the purified enzyme, 5 mM MgCl_2_, and increased concentrations of *sn*-glycerol 3-phosphate bis(cyclohexylammonium) salt (Sigma-Aldrich) in Tris-HCl (50 mM, pH 7.0). The samples were withdrawn and the released inorganic phosphate was analyzed as previously described (44). One unit (U) of G3PP activity was defined as the amount of enzyme that produced 1 μmol of inorganic phosphate per minute.

### Analytical methods.

The concentrations of succinate and glycerol were determined using a high-performance liquid chromatography (HPLC) LC-20AT system (Shimadzu, Japan) that was equipped with an Aminex HPX-87H column (300 × 7.8 mm, Bio-Rad, USA) and a refractive index detector. Briefly, the samples were boiled at 105°C for 15 min and centrifuged at 14,000 × *g* for 15 min. The supernatants were analyzed by using 10 mM H_2_SO_4_ as the mobile phase with a flow rate of 0.4 mL min^−1^. The concentration of glucose was determined by using a bio-analyzer (SBA-40D; Shandong Academy of Sciences).

### Data availability.

The raw data are publicly available through the NCBI SRA database (SRA accession number: PRJNA862672).
