# Supplementary figures and images for: Metabolic Mechanism and Physiological Role of Glycerol 3-Phosphate in Pseudomonas aeruginosa PAO1
Source: mBio. 2022 Oct 11;13(6):e02624-22. doi: 10.1128/mbio.02624-22 (PMC9765544; doi:10.1128/mbio.02624-22)

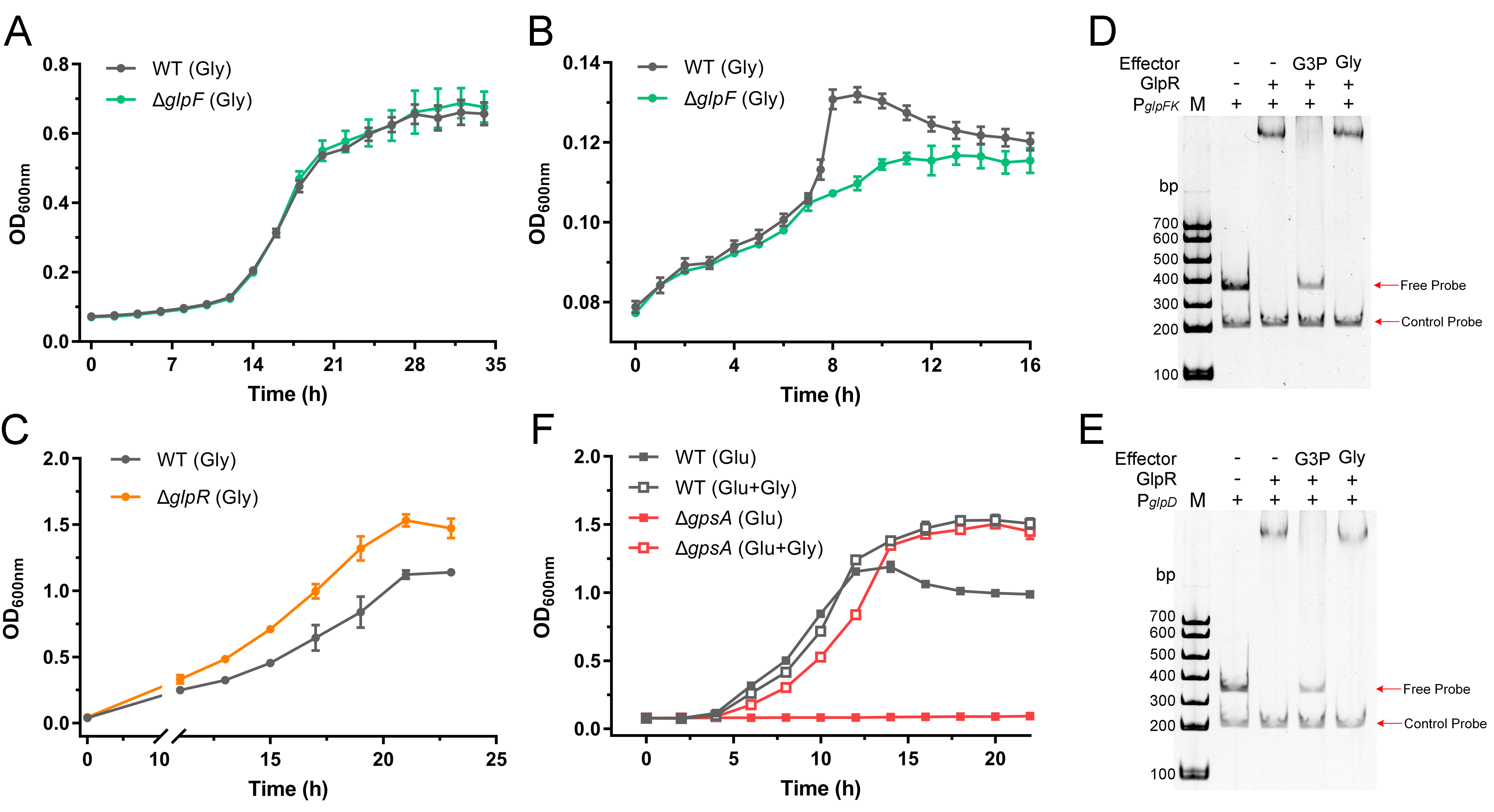

Supplement: FIG S1 [file mbio.02624-22-s0001.tif]

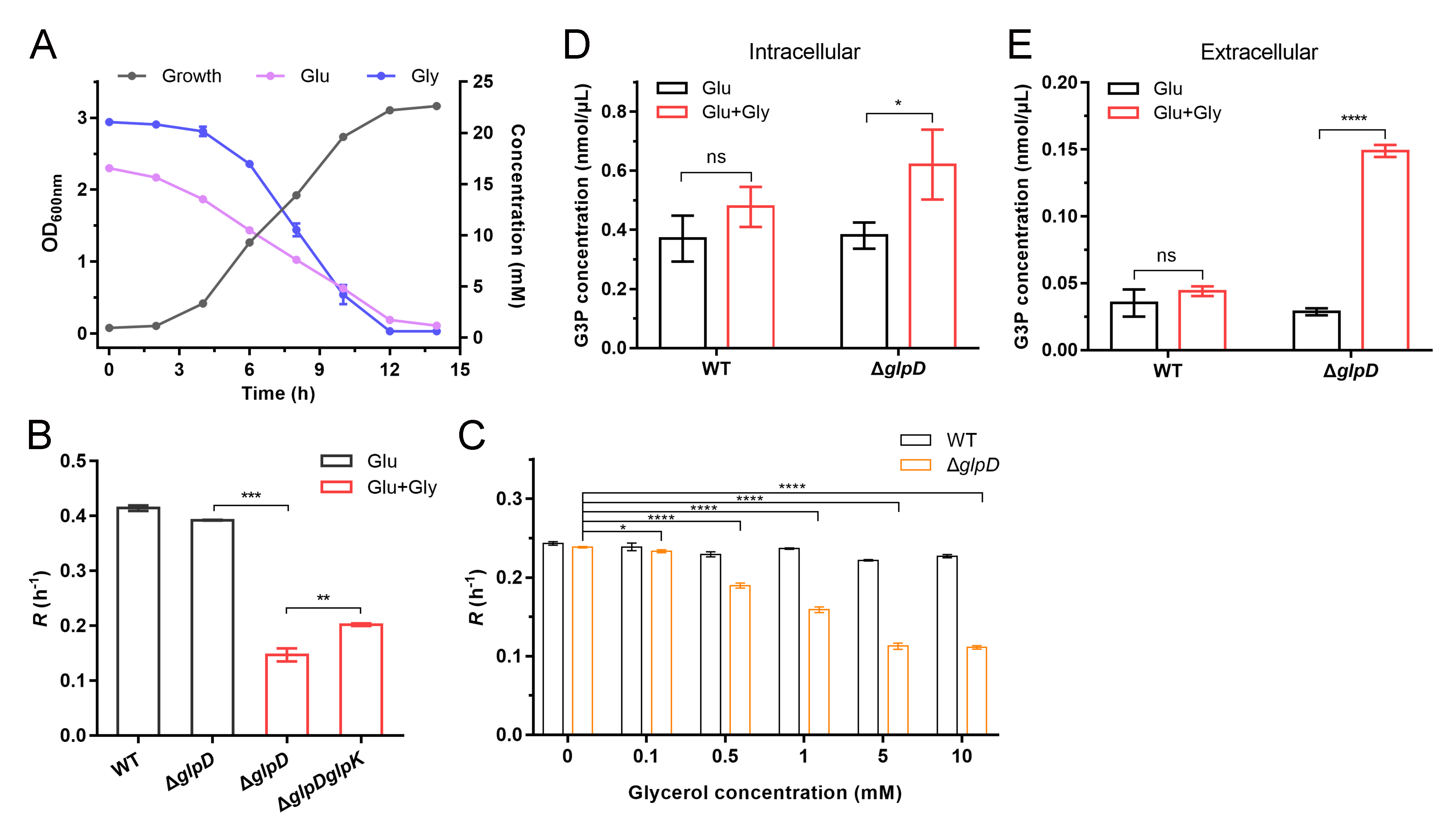

Supplement: FIG S2 [file mbio.02624-22-s0002.tif]

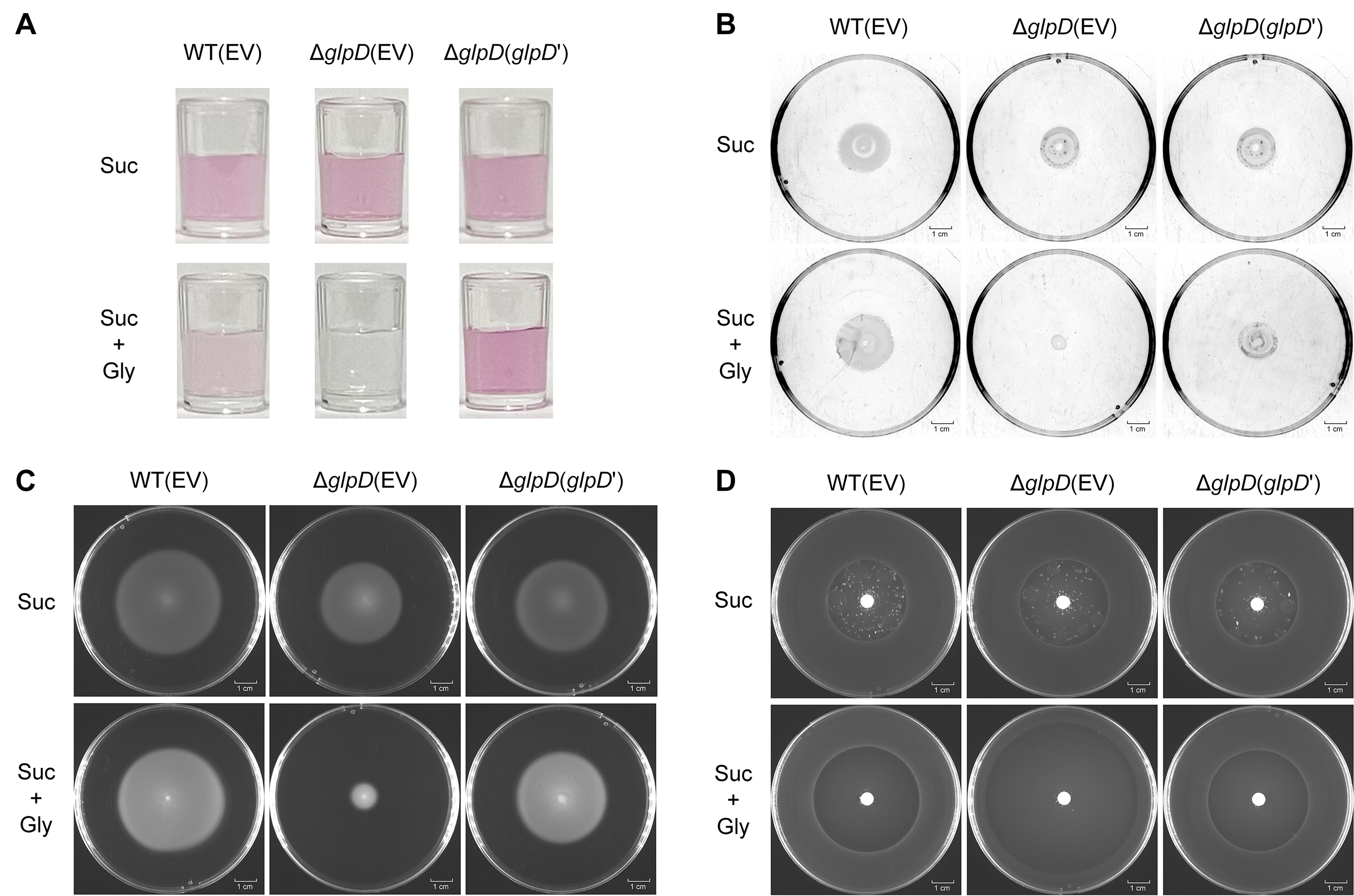

Supplement: FIG S3 [file mbio.02624-22-s0003.tif]

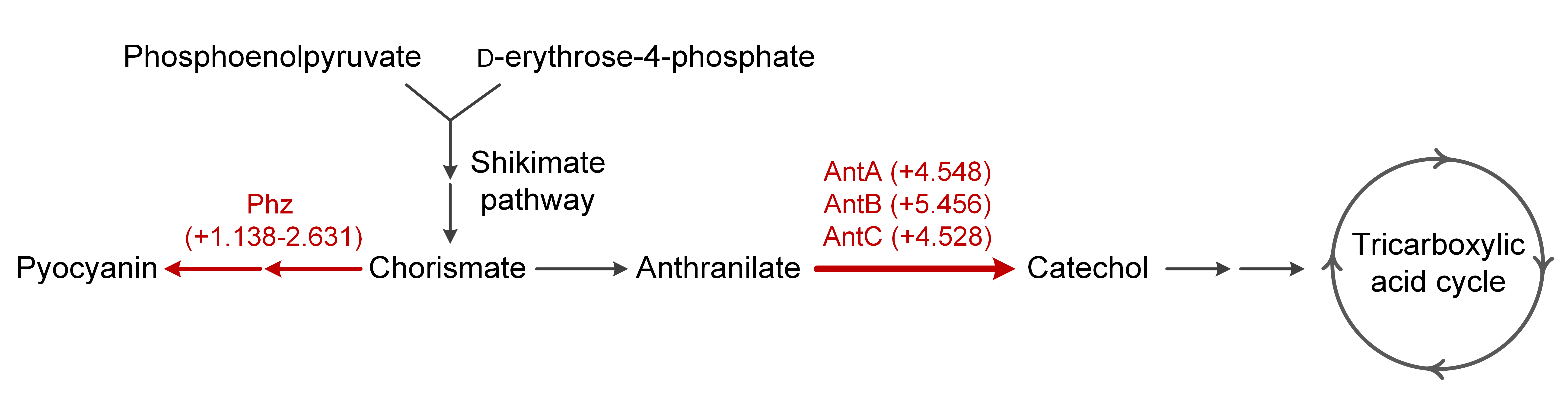

Supplement: FIG S4 [file mbio.02624-22-s0004.tif]

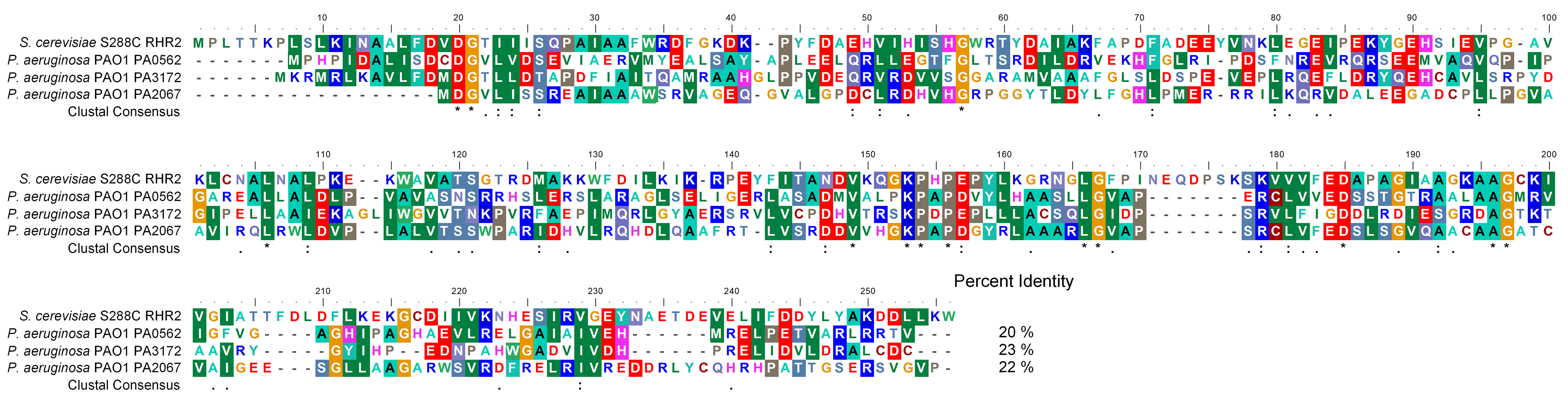

Supplement: FIG S5 [file mbio.02624-22-s0005.tif]

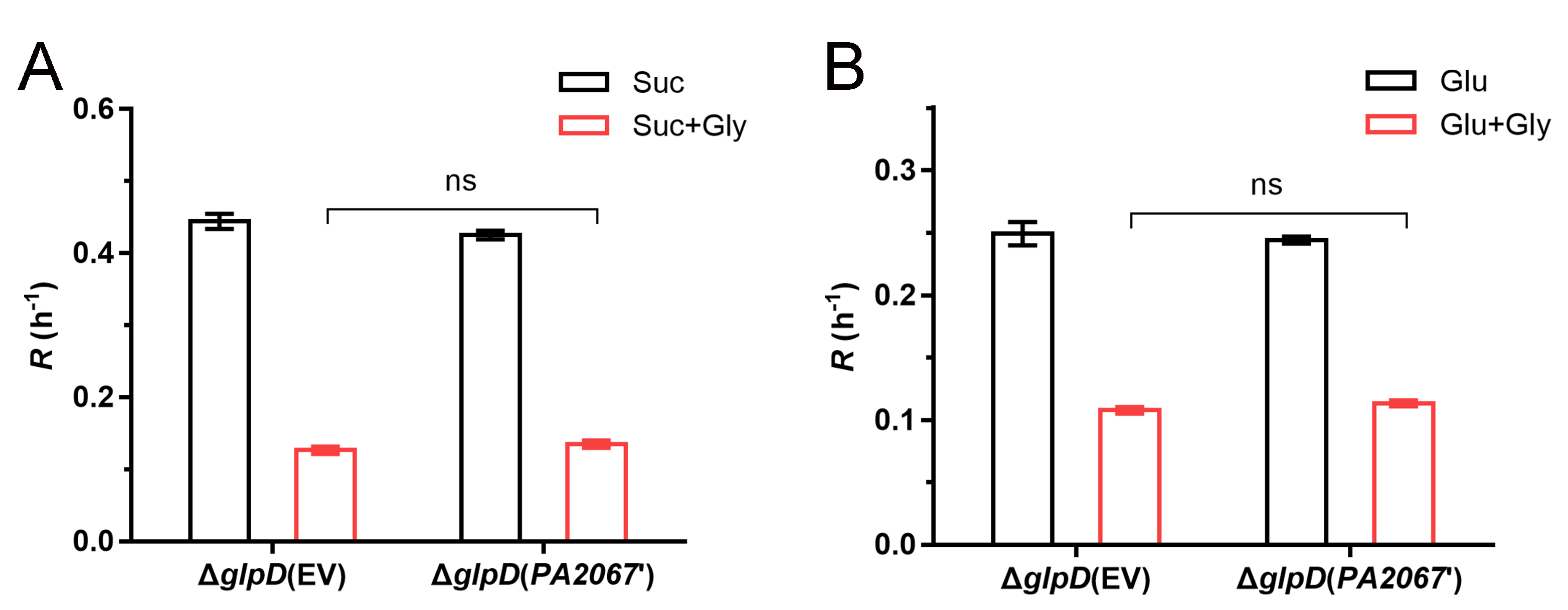

Supplement: FIG S6 [file mbio.02624-22-s0006.tif]
