# Supplementary material for: Metabolic Mechanism and Physiological Role of Glycerol 3-Phosphate in Pseudomonas aeruginosa PAO1
Source: mBio. 2022 Oct 11;13(6):e02624-22. doi: 10.1128/mbio.02624-22 (PMC9765544; doi:10.1128/mbio.02624-22)
Supplement: TABLE S1 [file mbio.02624-22-s0007.doc]

**TABLE S1** **Fold changes of mRNA transcripts in *P. aeruginosa* PAO1 (Δ*glpD*) under G3P stress**

| **Gene name** | **Locus_tag** | **DG vs DS^a^**  **(Log_2_ FC)** | ***P* value** | **Description** |
| --- | --- | --- | --- | --- |
| **Anthranilate catabolism** | | | | |
| *antA* | PA2512 | 4.548 | 1.10E-28 | anthranilate dioxygenase large subunit |
| *antB* | PA2513 | 5.456 | 2.71E-19 | anthranilate dioxygenase small subunit |
| *antC* | PA2514 | 4.528 | 3.92E-27 | anthranilate dioxygenase reductase |
| **Pyocyanin biosynthesis** | | | | |
| *phzA1* | PA4210 | 2.494 | 1.52E-12 | phenazine biosynthesis protein |
| *phzB1* | PA4211 | 1.946 | 5.49E-10 | phenazine biosynthesis protein |
| *phzC1* | PA4212 | 1.401 | 1.19E-05 | phenazine biosynthesis protein |
| *phzD1* | PA4213 | 1.763 | 1.74E-08 | phenazine biosynthesis protein |
| *phzE1* | PA4214 | 1.673 | 9.58E-08 | phenazine biosynthesis protein |
| *phzF1* | PA4215 | 1.138 | 7.25E-04 | trans-2,3-dihydro-3-hydroxyanthranilate isomerase |
| *phzG1* | PA4216 | 1.965 | 2.83E-10 | pyridoxamine 5'-phosphate oxidase |
| *phzA2* | PA1899 | 1.994 | 5.20E-08 | phenazine biosynthesis protein |
| *phzB2* | PA1900 | 1.804 | 2.62E-08 | phenazine biosynthesis protein |
| *phzC2* | PA1901 | 1.373 | 1.88E-05 | phenazine biosynthesis protein |
| *phzD2* | PA1902 | 1.763 | 1.74E-08 | phenazine biosynthesis protein |
| *phzE2* | PA1903 | 1.673 | 9.58E-08 | phenazine biosynthesis protein |
| *phzF2* | PA1904 | 1.138 | 7.25E-04 | trans-2,3-dihydro-3-hydroxyanthranilate isomerase |
| *phzG2* | PA1905 | 1.885 | 1.61E-09 | pyridoxamine 5'-phosphate oxidase |
| *phzH* | PA0051 | 2.631 | 6.02E-15 | phenazine-modifying protein |
| *phzS* | PA4217 | 1.280 | 1.08E-04 | flavin-containing monooxygenase |
| *phzM* | PA4209 | 1.786 | 1.84E-08 | phenazine-specific methyltransferase |
| **Type IVa pili biogenesis** | | | | |
| *pilA* | PA4525 | -4.190 | 1.23E-48 | type IVa pili protein |
| *pilB* | PA4526 | -2.558 | 2.52E-23 | type IVa pili biogenesis protein |
| *pilD* | PA4528 | -2.239 | 7.36E-19 | type IVa prepilin peptidase |
| *pilQ* | PA5040 | -1.216 | 4.81E-08 | type IVa pili biogenesis outer membrane protein |
| *pilP* | PA5041 | -1.252 | 2.84E-08 | type IVa pili biogenesis protein |
| *pilO* | PA5042 | -1.329 | 5.04E-09 | type IVa pili biogenesis protein |
| *pilN* | PA5043 | -1.473 | 2.09E-10 | type IVa pili biogenesis protein |
| *pilM* | PA5044 | -1.469 | 2.21E-10 | type IVa pili biogenesis protein |
| **Oxidative stress** | | | | |
| *oxyR* | PA5344 | -1.520 | 9.34E-11 | transcriptional regulator |
| *lsfA* | PA3450 | -2.217 | 1.37E-18 | 1-Cys peroxiredoxin |
| **Sulfate metabolism** | | | | |
| *cysA* | PA0280 | -1.704 | 9.37E-13 | sulfate.thiosulfate ABC transporter ATP-binding protein |
| *cysW* | PA0281 | -1.621 | 1.16E-11 | sulfate transporter |
| *cysT* | PA0282 | -1.737 | 8.68E-13 | sulfate transporter |
| *sbp* (*cysP*) | PA0283 | -1.852 | 2.53E-14 | sulfate-binding protein |
| *cysN* | PA4442 | -1.449 | 3.99E-10 | bifunctional sulfate adenylyltransferase subunit 1/adenylylsulfate kinase |
| *cysD* | PA4443 | -1.906 | 1.64E-14 | sulfate adenylyltransferase subunit 2 |
| **Taurine metabolism** | | | | |
| *tauA* | PA3936 | -0.968 | 8.20E-06 | taurine ABC transporter permease |
| *tauB* | PA3937 | -1.487 | 3.54E-10 | taurine ABC transporter ATP-binding protein |
| *tauC* | PA3938 | -1.437 | 8.53E-10 | taurine-binding protein |
| *tauD* | PA3935 | -0.861 | 4.53E-05 | taurine dioxygenase |
| **Alkanesulfonate metabolism** | | | | |
| *ssuA* | PA3445 | -3.624 | 2.81E-38 | sulfonate ABC transporter substrate-binding protein |
| *ssuB* | PA3442 | -2.188 | 1.62E-17 | aliphatic sulfonates ABC transporter ATP-binding subunit |
| *ssuC* | PA3443 | -2.685 | 3.30E-24 | ABC transporter permease |
| *ssuD* | PA3444 | -3.499 | 2.53E-36 | alkanesulfonate monooxygenase |
| *ssuE* | PA3446 | -1.790 | 3.21E-13 | NAD(P)H-dependent FMN reductase |
| **Iron-sulfur cluster biosynthesis** | | | | |
| *iscR* | PA3815 | -3.038 | 3.19E-29 | HTH-type transcriptional regulator |
| *iscS* | PA3814 | -2.575 | 1.12E-23 | cysteine desulfurase |
| *iscU* | PA3813 | -2.018 | 2.98E-16 | scaffold protein |
| *iscA* | PA3812 | -2.346 | 2.80E-20 | iron-binding protein |
| *hscB* | PA3811 | -2.137 | 1.08E-17 | co-chaperone |
| *hscA* | PA3810 | -1.578 | 1.47E-11 | chaperone protein |
| *fdx2* | PA3809 | -1.506 | 1.73E-10 | (2Fe-2S) ferredoxin |
| *iscX* | PA3808 | -0.433 | 9.65E-03 | Fe-S assembly protein |
| **Aerobic glycolysis** | | | | |
| *zwf* | PA3183 | -1.831 | 3.77E-14 | glucose-6-phosphate 1-dehydrogenase |
| *pgl* | PA3182 | -1.068 | 2.20E-06 | 6-phosphogluconolactonase |
| *eda* | PA3181 | -0.847 | 7.10E-05 | 2-keto-3-deoxy-6-phosphogluconate aldolase |
| *edd* | PA3194 | -1.153 | 1.67E-07 | 6-phosphogluconate dehydratase |
| *gapA* | PA3195 | -1.778 | 2.17E-13 | glyceraldehyde 3-phosphate dehydrogenase |
| *aceE* | PA5015 | -2.190 | 2.01E-18 | pyruvate dehydrogenase complex component E1 |
| *aceF* | PA5016 | -2.043 | 1.18E-16 | pyruvate dehydrogenase complex component E2 |
| **Terminal oxidase** | | | | |
| *cyoA* | PA1317 | -2.547 | 1.30E-22 | cytochrome o ubiquinol oxidase subunit II |
| *cyoB* | PA1318 | -2.056 | 1.12E-16 | cytochrome o ubiquinol oxidase subunit I |
| *cyoC* | PA1319 | -1.688 | 2.85E-12 | cytochrome o ubiquinol oxidase subunit III |
| *cyoD* | PA1320 | -1.707 | 3.27E-12 | cytochrome o ubiquinol oxidase subunit IV |
| *cyoE* | PA1321 | -1.598 | 1.83E-11 | protoheme IX farnesyltransferase |
| **Nitrate and nitrite transport** | | | | |
| *narK1* | PA3877 | 2.724 | 1.76E-14 | nitrite extrusion protein 1 |
| *narK2* | PA3876 | 3.313 | 8.66E-22 | nitrite extrusion protein 2 |
| **Nitrate reductase** | | | | |
| *narG* | PA3875 | 3.731 | 1.73E-28 | respiratory nitrate reductase subunit alpha |
| *narH* | PA3874 | 2.883 | 3.03E-17 | respiratory nitrate reductase subunit beta |
| *narJ* | PA3873 | 3.255 | 1.64E-17 | respiratory nitrate reductase subunit delta |
| *narI* | PA3872 | 2.321 | 1.32E-09 | respiratory nitrate reductase subunit gamma |
| **Nitrite reductase** | | | | |
| *nirS* | PA0519 | 1.902 | 1.63E-08 | nitrite reductase |
| *nirM* | PA0518 | 1.891 | 3.98E-06 | cytochrome C-551 |
| *nirC* | PA0517 | 2.790 | 2.96E-11 | cytochrome c55X |
| *nirF* | PA0516 | 3.362 | 3.85E-21 | heme d1 biosynthesis protein |
| *nirD* | PA0515 | 3.219 | 8.26E-15 | heme d1 biosynthesis protein |
| *nirL* | PA0514 | 3.205 | 1.58E-15 | heme d1 biosynthesis protein |
| *nirG* | PA0513 | 4.525 | 1.12E-22 | heme d1 biosynthesis protein |
| *nirH* | PA0512 | 4.328 | 1.12E-22 | heme d1 biosynthesis protein |
| *nirJ* | PA0511 | 3.386 | 1.32E-21 | heme d1 biosynthesis protein |
| *nirE* | PA0510 | 2.727 | 5.34E-13 | uroporphyrin-III C-methyltransferase |
| *nirN* | PA0509 | 3.432 | 4.75E-22 | cytochrome C |
| **Nitrous oxide reductase** | | | | |
| *nosR* | PA3391 | 4.731 | 5.31E-36 | regulatory protein NosR |
| *nosZ* | PA3392 | 2.844 | 7.73E-15 | nitrous-oxide reductase |
| *nosD* | PA3393 | 3.702 | 1.19E-20 | copper-binding periplasmic protein |
| *nosF* | PA3394 | 5.388 | 1.45E-30 | copper ABC transporter ATP-binding protein |
| *nosY* | PA3395 | 4.157 | 1.17E-22 | membrane protein |
| *nosL* | PA3396 | 3.965 | 3.85E-19 | acessory protein |
| *nosL* | PA3396 | 3.965 | 3.85E-19 | acessory protein |

^a^Fold change of gene expression after glycerol treatment compared with that of before glycerol treatment in *P. aeruginosa* PAO1 (Δ*glpD*).
