# Supplementary material for: Metabolic Mechanism and Physiological Role of Glycerol 3-Phosphate in Pseudomonas aeruginosa PAO1
Source: mBio. 2022 Oct 11;13(6):e02624-22. doi: 10.1128/mbio.02624-22 (PMC9765544; doi:10.1128/mbio.02624-22)
Supplement: TABLE S2 [file mbio.02624-22-s0008.doc]

**Table S2. Strains and plasmids used in this study**

| **Strain or plasmid** | **Relevant characteristics^a^** |
| --- | --- |
| **Strain** | |
| *P. aeruginosa* PAO1 | Wild-type |
| *P. aeruginosa* PAO1 (Δg*lpF*) | *P. aeruginosa* PAO1 mutant obtained by deletion of the *glpF* gene |
| *P. aeruginosa* PAO1 (Δg*lpK*) | *P. aeruginosa* PAO1 mutant obtained by deletion of the *glpK* gene |
| *P. aeruginosa* PAO1 (Δg*lpR*) | *P. aeruginosa* PAO1 mutant obtained by deletion of the *glpR* gene |
| *P. aeruginosa* PAO1 (Δg*lpD*) | *P. aeruginosa* PAO1 mutant obtained by deletion of the *glpD* gene |
| *P. aeruginosa* PAO1 (Δg*lpT*) | *P. aeruginosa* PAO1 mutant obtained by deletion of the *glpT* gene |
| *P. aeruginosa* PAO1 (Δ*gpsA*) | *P. aeruginosa* PAO1 mutant obtained by deletion of the *gpsA* gene |
| *P. aeruginosa* PAO1 (Δg*lpF*) | *P. aeruginosa* PAO1 mutant obtained by deletion of the *glpF* gene |
| *P. aeruginosa* PAO1 (Δg*lpD*Δ*glpK*) | *P. aeruginosa* PAO1 mutant obtained by deletion of the *glpD* and *glpK* gene |
| *P. aeruginosa* PAO1 (Δg*lpD*Δ*PA0562*Δ*PA3172*) | *P. aeruginosa* PAO1 mutant obtained by deletion of the *glpD*, *PA0562*, and *PA3172* gene |
| HB101 | *ara14 proA2 lacY1 galK2 xy15 λ*^-^ *mtl1 rpsL20(*Sm^r^*) glnV44 λ^-^*; triparental mating helper strain |
| *P. aeruginosa* PAO1 (EV) | *P. aeruginosa* PAO1 harboring the plasmid pBBR1MCS-5 |
| *P. aeruginosa* PAO1 (Δ*glpD*) (EV) | *P. aeruginosa* PAO1 (Δ*glpD*) harboring the plasmid pBBR1MCS-5 |
| *P. aeruginosa* PAO1 (Δ*glpD*) (*glpD*’) | *P. aeruginosa* PAO1 (Δ*glpD*) harboring the plasmid pBBR-*glpD* |
| *P. aeruginosa* PAO1 (Δ*glpD*) (*PA2067*’) | *P. aeruginosa* PAO1 (Δ*glpD*) harboring the plasmid pBBR-*PA2067* |
| *P. aeruginosa* PAO1 (Δ*glpD*) (*PA0562*’) | *P. aeruginosa* PAO1 (Δ*glpD*) harboring the plasmid pBBR-*PA0562* |
| *P. aeruginosa* PAO1 (Δ*glpD*) (*PA3172*’) | *P. aeruginosa* PAO1 (Δ*glpD*) harboring the plasmid pBBR-*PA3172* |
| *E. coli* BL21(DE3) | F^–^ *ompT hsdSB*(*rB- mB-*) *gal*(λ *c I* 857 *ind1 Sam*7 *nin*5 *lac*UV5-T7*gene*1) *dcm* (DE3) |
| BL21-*PA0562* | *E. coli* BL21(DE3) harboring the expression plasmid pETDuet-*PA0562* |
| BL21-*PA3172* | *E. coli* BL21(DE3) harboring the expression plasmid pETDuet-*PA3172* |
| *E. coli* DH5α | F^–^ φ80*lacZ*∆M15 ∆(*lacZYA-argF*)U169 *deoR recA*1 *endA*1 *hsdR*17(r_K_^–^, m_K_^+^) *phoA* *supE*44 λ^–^ *thi-*1 *gyrA*96 *relA*1, used for gene clone |
| **Plasmid** | |
| pK18*mobsacB-tet* | Suicide plasmid for gene knockout; Km^r^ and Tc^r^ |
| pK18*mobsacB*-Δ*glpF* | Partial lengths of *glpF* were inserted into pK18*mobsacB-tet* |
| pK18*mobsacB*-Δ*glpK* | Partial lengths of *glpK* were inserted into pK18*mobsacB-tet* |
| pK18*mobsacB*-Δ*glpR* | Partial lengths of *glpR* were inserted into pK18*mobsacB-tet* |
| pK18*mobsacB*-Δ*glpD* | Partial lengths of *glpD* were inserted into pK18*mobsacB-tet* |
| pK18*mobsacB*-Δ*glpT* | Partial lengths of *glpT* were inserted into pK18*mobsacB-tet* |
| pK18*mobsacB*-Δ*gpsA* | Partial lengths of *gpsA* were inserted into pK18*mobsacB-tet* |
| pK18*mobsacB*-Δ*PA0562* | Partial lengths of *PA0562* were inserted into pK18*mobsacB-tet* |
| pK18*mobsacB*-Δ*PA3172* | Partial lengths of *PA3172* were inserted into pK18*mobsacB-tet* |
| pRK2013 | Co1E1 ori, Km^r^, *mob*, *tra*^+^, helper plasmid for conjugation experiments |
| pBBR1MCS-5 | broad host range cloning vector; Gm^r^ |
| pBBR-*glpD* | pBBR1MCS-5 carrying *glpD* gene |
| pBBR-*PA2067* | pBBR1MCS-5 carrying *PA2067* gene |
| pBBR-*PA0562* | pBBR1MCS-5 carrying *PA0562* gene |
| pBBR-*PA3172* | pBBR1MCS-5 carrying *PA3172* gene |
| pETDuet-1 | Vector for protein expression; Ap^r^ |
| pETDuet-*glpR* | pETDuet-1 contained *glpR* gene of *P. aeruginosa* PAO1 |
| pETDuet-*PA0562* | pETDuet-1 contained *PA0562* gene of *P. aeruginosa* PAO1 |
| pETDuet-*PA3172* | pETDuet-1 contained *PA3172* gene of *P. aeruginosa* PAO1 |

^a^Ap^r^, ampicillin resistant; Km^r^, kanamycin resistant; Tc^r^, tetracycline resistant; Gm^r^, gentamicin resistant.
