# Supplementary material for: Metabolic Mechanism and Physiological Role of Glycerol 3-Phosphate in Pseudomonas aeruginosa PAO1
Source: mBio. 2022 Oct 11;13(6):e02624-22. doi: 10.1128/mbio.02624-22 (PMC9765544; doi:10.1128/mbio.02624-22)
Supplement: TABLE S3 [file mbio.02624-22-s0009.doc]

**Table S3. Primers used in this study**

| **Primer** | **Sequence(5’-3’)^a^** | **Use** |
| --- | --- | --- |
| **Gene knockout** | | |
| *glpF*-uf | CTTGAATTCTCACGGCTGCCCGCCATAA (EcoRI) | Amplification of upstream homologous arm of *glpF* (forward) |
| *glpF*-ur | CGGCTCGGCTTCGGCGGGTGATGTAGAACGGCAG | Amplification of upstream homologous arm of *glpF* (reverse) |
| *glpF*-df | CTGCCGTTCTACATCACCCGCCGAAGCCGAGCCG | Amplification of downstream homologous arm of *glpF* (forward) |
| *glpF*-dr | CATGGATCCGCCCGTCGCGCTTGAGCT (BamHI) | Amplification of downstream homologous arm of *glpF* (reverse) |
| *glpK*-uf | CTGGAATTCATGACCGACAAGCACAA (EcoRI) | Amplification of upstream homologous arm of *glpK* (forward) |
| *glpK*-ur | CATAGGGGTCCCAATAGGCCCGTCGCGCTTGAGCTGC | Amplification of upstream homologous arm of *glpK* (reverse) |
| *glpK*-df | AGCTCAAGCGCGACGGGCCTATTGGGACCCCTATG | Amplification of downstream homologous arm of *glpK* (forward) |
| *glpK*-dr | CTTGGATCCGTCCCAGCCGCGAGTGC (BamHI) | Amplification of downstream homologous arm of *glpK* (reverse) |
| *glpR*-uf | CTTGAATTCCTGGGTAACGTTTGCG (EcoRI) | Amplification of upstream homologous arm of *glpR* (forward) |
| *glpR*-ur | GAGTCGGCGGCCAGGAACAGCATGGTGTAGGCGGTG | Amplification of upstream homologous arm of *glpR* (reverse) |
| *glpR*-df | CACCGCCTACACCATGCTGTTCCTGGCCGCCGACTC | Amplification of downstream homologous arm of *glpR* (forward) |
| *glpR*-dr | CTAGGATCCACCAGCCCGATTCACCAG (BamHI) | Amplification of downstream homologous arm of *glpR* (reverse) |
| *glpD*-uf | CTTGAATTCTGGTCCGCGAAGCCCTG (EcoRI) | Amplification of upstream homologous arm of *glpD* (forward) |
| *glpD*-ur | CGTCAGCGCCGATTCGCAGGTGCCAGAGTCCC | Amplification of upstream homologous arm of *glpD* (reverse) |
| *glpD*-df | GGGACTCTGGCACCTGCGAATCGGCGCTGACG | Amplification of downstream homologous arm of *glpD* (forward) |
| *glpD*-dr | CTTGGATCCATGCTCGCTCTGCAGGTA (BamHI) | Amplification of downstream homologous arm of *glpD* (reverse) |
| *glpT*-uf | CTTGAATTCTTTCGTCCCGCGCCACACC (EcoRI) | Amplification of upstream homologous arm of *glpT* (forward) |
| *glpT*-ur | ACCAGGATCATGAACACCAGTAGCCCAGTGCGCGAAT | Amplification of upstream homologous arm of *glpT* (reverse) |
| *glpT*-df | GATTCGCGCACTGGGCTACTGGTGTTCATGATCCTGGT | Amplification of downstream homologous arm of *glpT* (forward) |
| *glpT*-dr | CTTGGATCCCGCAGGGTCAGGCAGAGG (BamHI) | Amplification of downstream homologous arm of *glpT* (reverse) |
| *gpsA*-uf | CTGGAATTCATGACAGAGCAGCAACCGAT (EcoRI) | Amplification of upstream homologous arm of *gpsA* (forward) |
| *gpsA*-ur | CCGAGGGCGTGGCCGACCGCAGGGCGCTGGAGGGCAA | Amplification of upstream homologous arm of *gpsA* (reverse) |
| *gpsA*-df | TTGCCCTCCAGCGCCCTGCGGTCGGCCACGCCCTCGG | Amplification of downstream homologous arm of *gpsA* (forward) |
| *gpsA*-dr | CTAGGATCCAGAAGCCGGTGGTGGGAATGAA (BamHI) | Amplification of downstream homologous arm of *gpsA* (reverse) |
| *PA0562-*uf | CTGGAATTCGCTGCCGGCCCTGGGCGTCACC (EcoRI) | Amplification of upstream homologous arm of *PA0562* (forward) |
| *PA0562-*ur | GCCCTGACTCAGACTGTCCGCATGGGCTACCTCGCTGC | Amplification of upstream homologous arm of *PA0562* (reverse) |
| *PA0562-*df | CGCAGCGAGGTAGCCCATGCGGACAGTCTGAGTCAGGGC | Amplification of downstream homologous arm of *PA0562* (forward) |
| *PA0562-*dr | CTAGGATCCACTCCATGGTCGATTTCTTCT (BamHI) | Amplification of downstream homologous arm of *PA0562* (reverse) |
| *PA3172-*uf | CTGGAATTCGATCCGCGCCTGCCACCGCCTG (EcoRI) | Amplification of upstream homologous arm of *PA3172* (forward) |
| *PA3172-*ur | TGTCGGCGGCGCTGCGGATCACCGCATGCGCTTCACGCCTCC | Amplification of upstream homologous arm of *PA3172* (reverse) |
| *PA3172-*df | GGAGGCGTGAAGCGCATGCGGTGATCCGCAGCGCCGCCGACA | Amplification of downstream homologous arm of *PA3172* (forward) |
| *PA3172-*dr | CTAGGATCCTGGTGCGGCTGGGCGGTCTC (BamHI) | Amplification of downstream homologous arm of *PA3172* (reverse) |
| **Gene overexpression** | | |
| *glpD*-F | CCCAAGCTTATGAGTCAAGCGCACACC (HindIII) | Amplification of *glpD* in *P. aeruginosa* PAO1 (forward) |
| *glpD*-R | ATTGGATCCTCAGGCCGCATGCACCCGC (BamHI) | Amplification of *glpD* in *P. aeruginosa* PAO1 (reverse) |
| *PA2067*-F | CCCAAGCTTATGGACGGCGTCCTGATCA (HindIII) | Amplification of *PA2067* in *P. aeruginosa* PAO1 (forward) |
| *PA2067*-R | CCGGAATTCTCATGGGACGCCAACGCTC (EcoRI) | Amplification of *PA2067* in *P. aeruginosa* PAO1 (reverse) |
| *PA0562*-F | CCCAAGCTTATGCCCCACCCCATCGACGC (HindIII) | Amplification of *PA0562* in *P. aeruginosa* PAO1 (forward) |
| *PA0562*-R | CCGGAATTCTCAGACTGTCCGGCGCAAGC (EcoRI) | Amplification of *PA0562* in *P. aeruginosa* PAO1 (reverse) |
| *PA3172*-F | CCCAAGCTTGTGAAGCGCATGCGGCTCAAAG (HindIII) | Amplification of *PA3172* in *P. aeruginosa* PAO1 (forward) |
| *PA3172*-R | CCGGAATTCTCAGCAGTCGCAGAGC (EcoRI) | Amplification of *PA3172* in *P. aeruginosa* PAO1 (reverse) |
| **Protein expression** | | |
| GlpR-F | CTGGAATTCGATGAACCTGCCCCCCCG (EcoRI) | Amplification of *glpR* in *P. aeruginosa* PAO1 (forward) |
| GlpR-R | CCCAAGCTTTCAGACCAGGTCCAGG (HindIII) | Amplification of *glpR* in *P. aeruginosa* PAO1 (reverse) |
| PA0562-F | CCGGAATTCGATGCCCCACCCCATCGACGC (EcoRI) | Amplification of *PA0562* in *P. aeruginosa* PAO1 (forward) |
| PA0562-R | CCCAAGCTTTCAGACTGTCCGGCGCAA (HindIII) | Amplification of *PA0562* in *P. aeruginosa* PAO1 (reverse) |
| PA3172-F | CCGGAATTCGGTGAAGCGCATGCGGCTCAAA (EcoRI) | Amplification of *PA3172* in *P. aeruginosa* PAO1 (forward) |
| PA3172-R | CCCAAGCTTTCAGCAGTCGCAGAG (HindIII) | Amplification of *PA3172* in *P. aeruginosa* PAO1 (reverse) |
| **EMSAs** | |  |
| P*_glpD_*-F | GGCATTTCCCCTGCCACGGCG | Amplification of *glpD* promoter fragment (forward) |
| P*_glpD_*-R | ACATCGTAGACTTCGGCGAGTGGAG | Amplification of *glpD* promoter fragment (reverse) |
| P*_glpFK_*-F | GGTCACTCCCGGAGAAGAACGT | Amplification of *glpFK* promoter fragment (forward) |
| P*_glpFK_*-R | TCGGCCAGGCATTGGCCGAACAGGG | Amplification of *glpFK* promoter fragment (reverse) |
| CP-F | CCTGGTGGTGCTCAACGC | Amplification of control probe (forward) |
| CP-R | GGGCGACTTCTGTTTCAGGT | Amplification of control probe (reverse) |

^a^Restriction sites are underlined, and the restriction enzymes are indicated in parentheses.
